# Chemotherapy agents stimulate dendritic cells against human colon cancer cells through upregulation of the transporter associated with antigen processing

**DOI:** 10.1038/s41598-021-88648-z

**Published:** 2021-04-27

**Authors:** Yi-Hsin Liang, Jia-Huei Tsai, Yung-Ming Cheng, Kuang-Yu Chan, Wen-Ling Hsu, Chang-Cheng Lee, Kuo-Hsing Chen, Kun-Huei Yeh

**Affiliations:** 1 Graduate Institute of Oncology, National Taiwan University, Taipei, Taiwan, R.O.C.; 2 Clinical Medicine, National Taiwan University, Taipei, Taiwan, R.O.C.; 3 Centers of Genomic and Precision Medicine, College of Medicine, National Taiwan University, Taipei, Taiwan, R.O.C.; 4 Department of Oncology, National Taiwan University Hospital, No 7, Chung-Shan South Rd, Taipei, 10002 Taiwan, R.O.C.; 5 Department of Pathology, National Taiwan University Hospital, Taipei, Taiwan, R.O.C.; 6 National Taiwan University Cancer Center, National Taiwan University Hospital, Taipei, Taiwan, R.O.C.

**Keywords:** Cancer, Cell biology, Immunology, Oncology

## Abstract

Single immunotherapy fails to demonstrate efficacy in patients with microsatellite stable (MSS) metastatic colorectal cancer (mCRC). Research on immune reactions before and after systemic agents for mCRC is warranted. Our study examined cell line models to compare the expression of immune surface markers on colon cancer cells before and after chemotherapy agents. We also elucidated mechanisms underlying the effects of chemotherapy agents on immune surface markers. We used real-world clinical samples with NanoString analysis and the Perkin-Elmer Opal multiplex system. We established that chemotherapy agents, particularly 7-ethyl-10-hydroxycamptothecin (SN-38), the active metabolite of irinotecan, stimulated the expression of stimulatory MHC class I alleles through stimulation the pathway of transporters associated with antigen processing 1 and 2 (TAP1 and TAP2) in cell line models. Application of infected cell protein 47 (ICP-47), a specific inhibitor of the TAP1/TAP2, significantly inhibited expression of TAP1/TAP2 and also inhibited the expression of the downstream MHC class I. In the functional assay, SN-38 significantly promoted the phagocytosis of colon cancer cells by monocyte-derived dendritic cells (MoDCs). We confirmed that the expression of major histocompatibility complex (MHC) class I, significantly increased after first-line chemotherapy and targeted therapy in the samples of real-world patients with de novo mCRC. Our study provides new insights for novel immunotherapy combinations.

## Introduction

Colorectal cancer (CRC) is both the third most prevalent cancer worldwide and the third leading cause of cancer death in Taiwan^1,2^. Currently, the treatment backbone of metastatic CRC (mCRC) continues to be chemotherapy and targeted agents^2–4^. Immunotherapy with immune checkpoint inhibitors (ICIs), such as anti-cytotoxic T lymphocyte–associated antigen 4 antibody and anti-programmed-death 1 (PD-1) antibodies, has demonstrated tremendous breakthroughs in treatments for melanoma, renal cell carcinoma, non–small-cell lung cancer, and several other cancer types^5–8^. By contrast, ICIs are applied only for patients with microsatellite instability high (MSI-H) mCRC^9,10^. However, MSI-H mCRC accounts for only 1.8–4.0% of all patients with mCRC^11^. A possible immune pathway must be surveyed to develop a new strategy for immunotherapy in mCRC treatment.

The immune microenvironment exerts effects on the efficacy and delivery of chemotherapy and targeted therapy^12,13^. Chemotherapy and targeted therapy might change the tumor microenvironment and immune surface markers^14–16^. Irinotecan increases the endoplasmic reticulum stress of tumor cells, subsequently inducing immunogenic cell death (ICD)^17^. Irinotecan also stimulated the expression of MHC class I on breast cancer model^18^. Oxaliplatin inhibits pSTAT6 in tumor cells, which subsequently inhibits the expression of PD-L2 and increases the expression of major histocompatibility complex (MHC) class I, namely human leukocyte antigen (HLA) class I. Both actions enhance the efficacy of cytotoxic T cells^19,20^. Moreover, oxaliplatin induces the expression of calreticulin in tumor cells and thus enhances ICD^16,20^. Fluorouacil (5-FU) stimulates the cytotoxicity of natural killer (NK) cell and also stimulates the expression of MHC class I and PD-L1 . Cetuximab, an antiepidermal growth factor receptor monoclonal antibody, can stimulate immune effector cells and induce antibody-dependent cell-mediated cytotoxicity (ADCC)^24,25^. Bevacizumab, an antivascular endothelial growth factor monoclonal antibody, targets endothelial cells in peritumor parts and exerts immune modulating effects by influencing the tumor microenvironment^26,27^. The aforementioned agents are all standard systemic agents for the treatment of mCRC. That is, research on immune reactions before and after systemic agents for mCRC is warranted for new immunotherapy targets.

Our study plans to incorporate data from real-world clinical samples into cell line models. We compared the expression of immune surface markers in colon cancer cells before and after chemotherapy agents and elucidated mechanisms underlying the effects of chemotherapy agents on immune surface markers. We would focus on the interactions between chemotherapy agents and antigen processing pathway and the subsequent dynamic change of MHC class I^28–30^.

## Results

### IFN-γ specifically stimulated the expression of stimulatory MHC class I alleles

**Figure 1:**
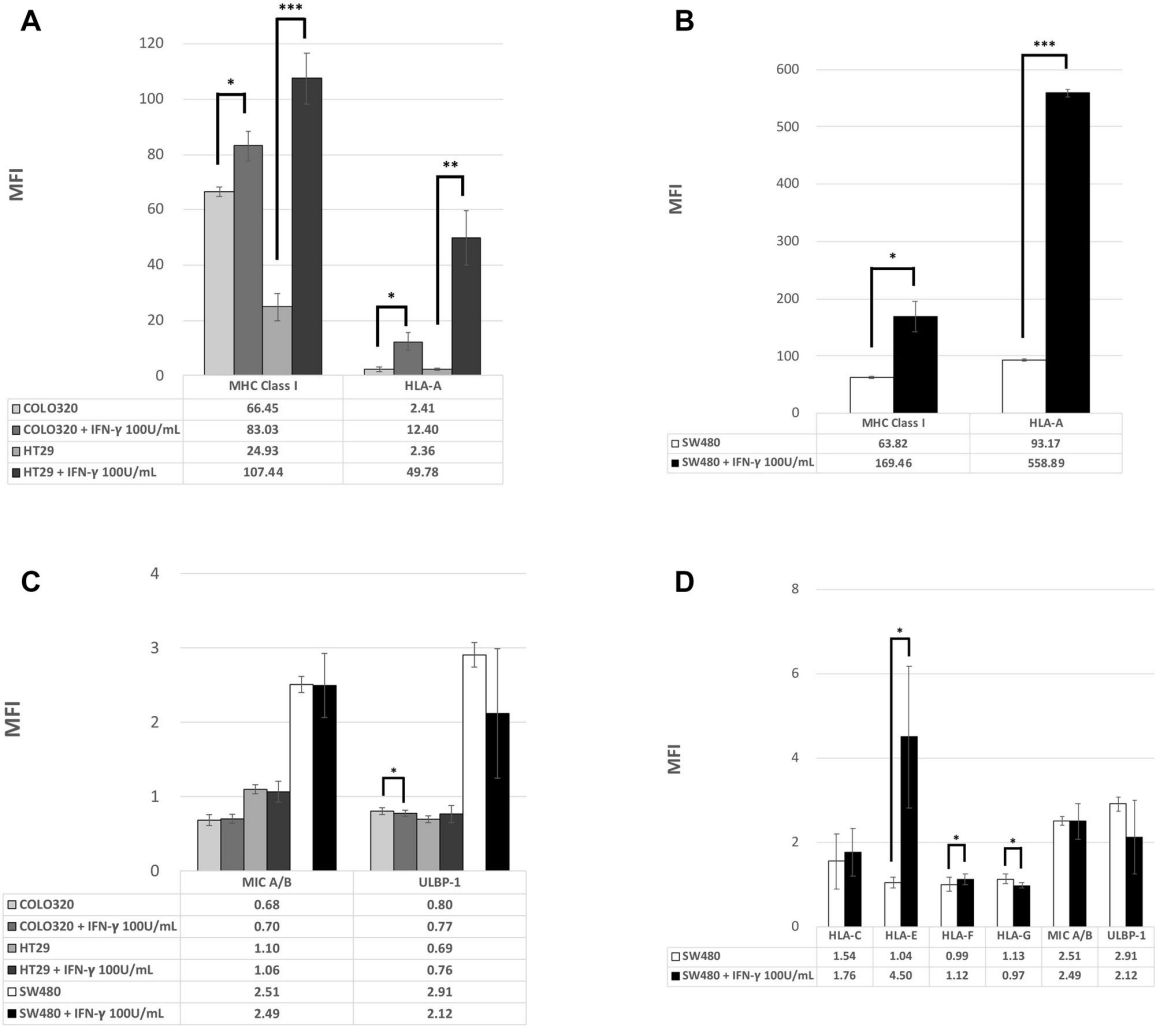
(**A**) Mean fluorescence intensity (MFI) for MHC class I and HLA-A expressed on two colon cell lines in response to IFN-γ after 48-h exposure. (*P < 0.05, **P < 0.01, ***P < 0.001). (**B**) Mean fluorescence intensity (MFI) for MHC class I and HLA-A expressed on SW480 cell lines in response to IFN-γ after 48-h exposure. (*P < 0.05, **P < 0.01, ***P < 0.001). (**C**) Mean fluorescence intensity (MFI) for NK cell ligands expressed on three colon cell lines in response to IFN-γ after 48-h exposure. (*P < 0.05, **P < 0.01, ***P < 0.001). (**D**) Mean fluorescence intensity (MFI) for MHC class I alleles and NK cell ligands expressed on SW480 cell lines in response to IFN-γ after 48-h exposure. (*P < 0.05, **P < 0.01, ***P < 0.001).

 First, we tested three colon cancer cell lines, SW480, COLO 320 and HT29, through flow cytometry. These cell lines all initially exhibited low expression of MHC class I and NK cell ligands. The expression of MHC class I significantly increased after IFN-γ stimulation (Fig. 1A,B). By contrast, NK cell ligands including MIC A/B and ULBP-1 were both irresponsive to IFN-γ (Fig. 1C). We then tested more allele expressions specifically on SW480. SW480 demonstrated low expression of all MHC class I alleles, including pan-MHC class I, HLA-A, HLA-C, HLA-E, HLA-F, and HLA-G. NK cell ligands, including MIC A/B and ULBP, both exhibited low expression for SW480 cells (Fig. 1B,D). IFN-γ specifically stimulated the expression of MHC class I, particularly HLA-A, but NK cell ligands were responsive to IFN-γ stimulation. In summary, IFN-γ significantly stimulates the expression of MHC class I, particularly HLA-A without stimulating NK cell ligands. Although it was non-significant, a mild trend occurred whereby the stimulatory effect of MHC class I and HLA-A in response to IFN-γ stimulation was positively correlated with IFN-γ dosage and incubation time.

### Chemotherapy agents, especially SN-38, which was the active metabolite of irinotecan, stimulated the expression of stimulatory MHC class I alleles and NK cell ligands

**Figure 2:**
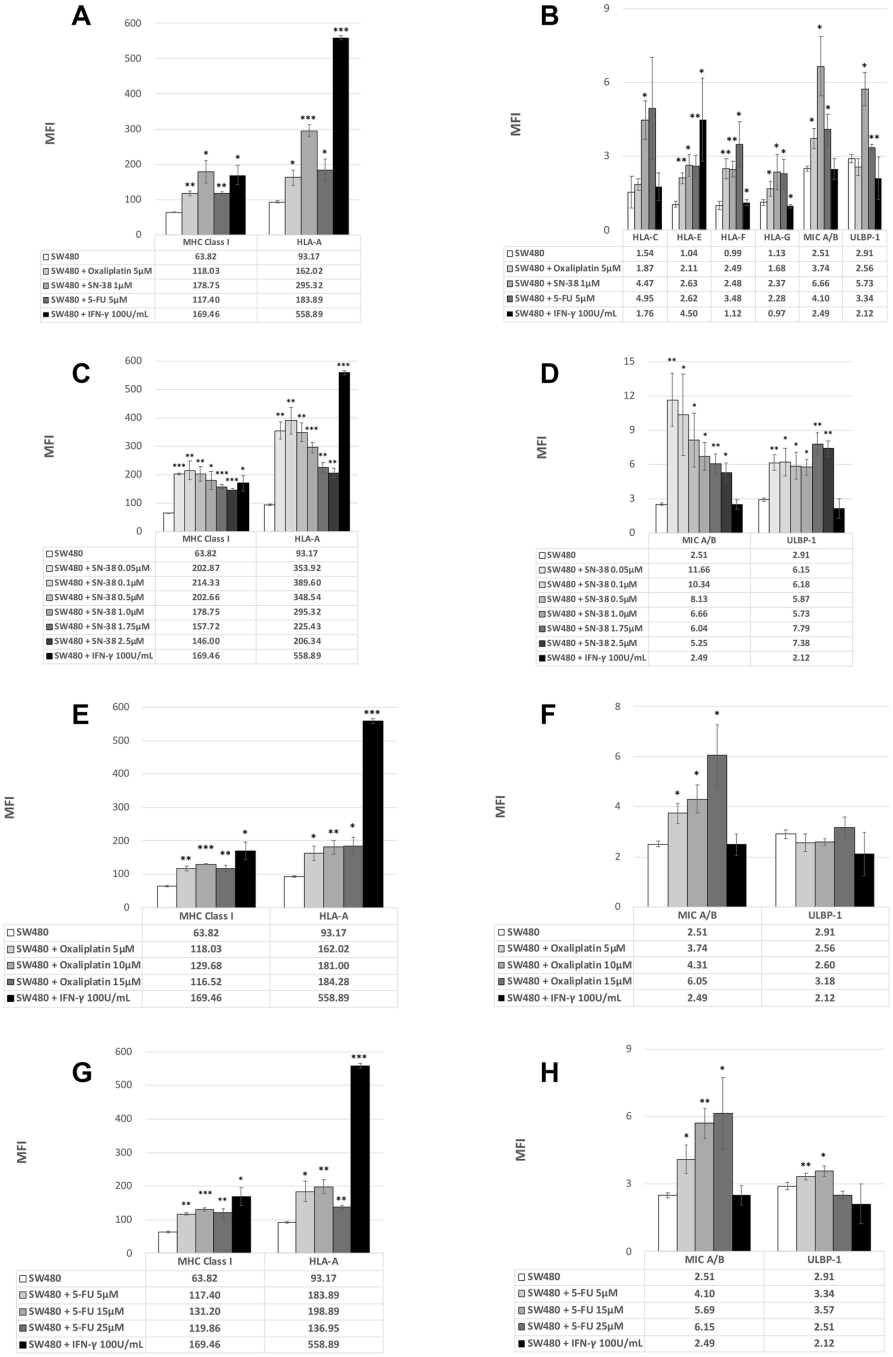
(**A**) Mean fluorescence intensity (MFI) for MHC class I and HLA-A expressed on SW480 cell lines in response to chemotherapy agents or IFN-γ (as a positive control). (*P < 0.05, **P < 0.01, ***P < 0.001). (**B**) Mean fluorescence intensity (MFI) for MHC class I alleles and NK cell ligands expressed on SW480 cell lines in response to chemotherapy agents or IFN-γ (as a positive control). (*P < 0.05, **P < 0.01, ***P < 0.001). (**C**) Mean fluorescence intensity (MFI) for MHC class I and HLA-A expressed on SW480 cell lines in response to SN-38 or IFN-γ after 48-h exposure. (*P < 0.05, **P < 0.01, ***P < 0.001). (**D**) Mean fluorescence intensity (MFI) for NK cell ligands expressed on SW480 cell lines in response to SN-38 or IFN-γ after 48-h exposure. (*P < 0.05, **P < 0.01, ***P < 0.001). (**E**) Mean fluorescence intensity (MFI) for MHC class I and HLA-A, expressed on SW480 cell lines in response to oxaliplatin or IFN-γ after 48-h exposure. (*P < 0.05, **P < 0.01, ***P < 0.001). (**F**) Mean fluorescence intensity (MFI) for NK cell ligands expressed on SW480 cell lines in response to oxaliplatin or IFN-γ after 48-h exposure. (*P < 0.05, **P < 0.01, ***P < 0.001). (**G**) Mean fluorescence intensity (MFI) for MHC class I and HLA-A expressed on SW480 cell lines in response to 5-FU or IFN-γ after 48-h exposure. (*P < 0.05, **P < 0.01, ***P < 0.001). (**H**) Mean fluorescence intensity (MFI) for NK cell ligands expressed on SW480 cell lines in response to 5-FU or IFN-γ after 48-h exposure. (*P < 0.05, **P < 0.01, ***P < 0.001).

 The chemotherapy backbone of mCRC consisted of 5-FU, oxaliplatin, and irinotecan. The cytotoxicity effect of irinotecan was exerted by the active metabolite SN-38. We firstly tested these three chemotherapy agents at the level of human pharmacokinetic studies (PK studies)^31^. Notably, all three of these chemotherapy agents increased the expression of MHC class I alleles, albeit at different levels (Fig. 2A,B). Unlike IFN-γ, SN-38, particularly, not only increased the expression of MHC class I but also increased the expression of NK cell ligands. Not only HLA-A, SN-38 also increased the expression of all MHC class I alleles. The dosage effect for these three agents greatly differed. SN-38 significantly increased the expression of surface markers even at low doses, and the stimulation declined gradually with higher doses (Fig. 2C,D). The peak stimulation effect on the SN-38 level of 0.1 μM demonstrated a high expression level comparable to the level stimulated by IFN-γ. Although the absolute fluorescence intensity (MFI) was relatively low after exposure to SN-38, the fold change of NK cell ligands was relatively high without the correlation to SN-38 concentration. By contrast, 5-FU and oxaliplatin only moderately stimulated the expression of MHC class I alleles at a peak level of 15 μM for 5-FU. The peak stimulation of pan-MHC class I for oxaliplatin was 10 μM, and the expression of HLA-A increased in combination with increasing oxaliplatin doses (Fig. 2E,F). However, extremely few tumor cells were viable with higher doses of oxaliplatin. Moreover, 5-FU and oxaliplatin stimulated the expression of NK cell ligands, although the expression remained extremely low (Fig. 2G,H).

### Chemotherapy agents, particularly SN-38, stimulated the expression of TAP1, TAP2, and tapasin, namely, the TAP pathway, and the subsequent MHC class I expression

**Figure 3:**
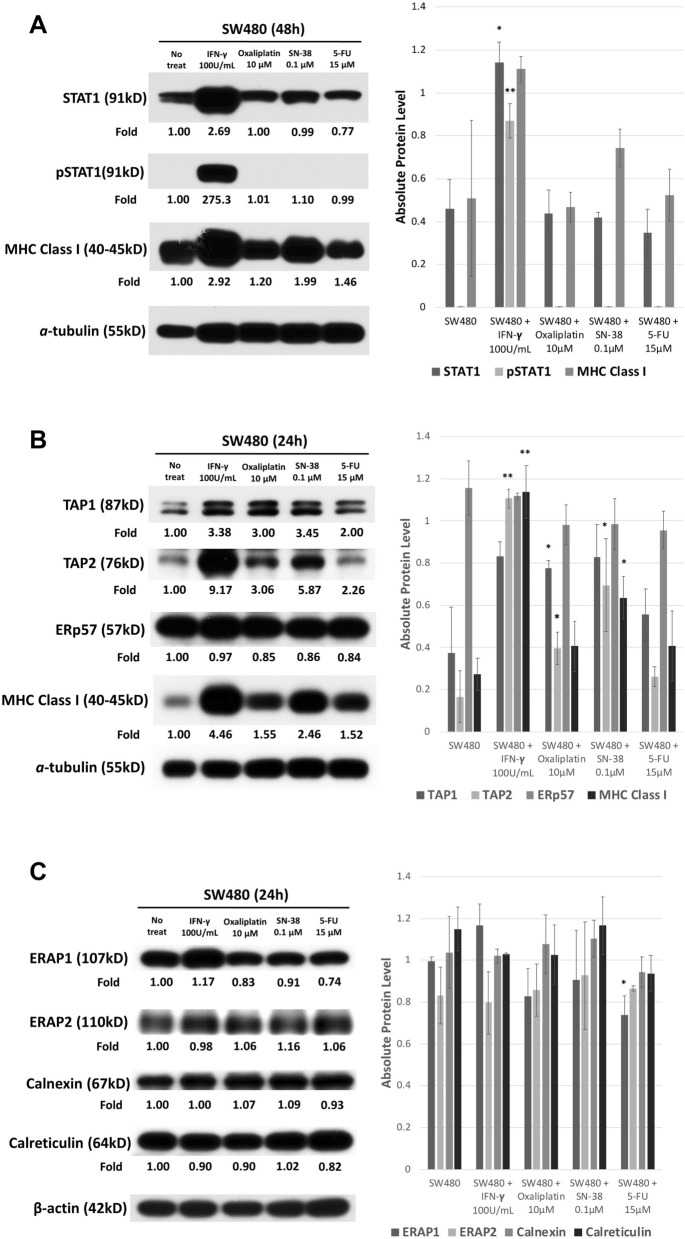
(**A**) Western blot for SW480 STAT1 pathway in response to chemotherapy agents. The full blot was displayed in Supplement figures. Photoshop was applied for only modifying brightness. ImageJ was applied for each signals to quantification. The results of quantification and statistical analysis were shown in the right side of this figure. Quantification of western blot for SW480 STAT1 pathway in response to chemotherapy agents by using ImageJ and statistical analysis by using SPSS with at least three or more independent tests. (*P < 0.05, **P < 0.01, ***P < 0.001). (**B**) Western blot for the SW480 antigen processing pathway in response to chemotherapy agents. The full blot was displayed in Supplement figures. Photoshop was applied for only modifying brightness. ImageJ was applied for each signals to quantification. The results of quantification and statistical analysis were shown in the right side of this figures. Quantification of western blot for the SW480 antigen processing pathway in response to chemotherapy agents by using ImageJ and statistical analysis by using SPSS with at least three or more independent tests. (*P < 0.05, **P < 0.01, ***P < 0.001). (**C**) Western blot for the SW480 antigen processing pathway in response to chemotherapy agents. The full blot was displayed in Supplement figures. Photoshop was applied for only modifying brightness. ImageJ was applied for each signals to quantification. The results of quantification and statistical analysis were shown in the right side of this figures. Quantification of western blot for the SW480 antigen processing pathway in response to chemotherapy agents by using ImageJ and statistical analysis by using SPSS with at least three or more independent tests. (*P < 0.05, **P < 0.01, ***P < 0.001).

 IFN-γ stimulated MHC class I expression by triggering the downstream pathway of STAT1, but none of these three chemotherapy agents stimulated the STAT1 pathway (Fig. 3A). We thus tested all other downstream antigen processing pathway molecules. Only TAP1 and TAP2 were significantly upregulated by chemotherapy agents, particularly SN-38. None of other molecules was stimulated by chemotherapy agents (Fig. 3B,C).

### ICP47, a specific TAP inhibitor, significantly inhibited expression of TAP1 and TAP2 as well as the expression of downstream MHC class I

**Figure 4:**
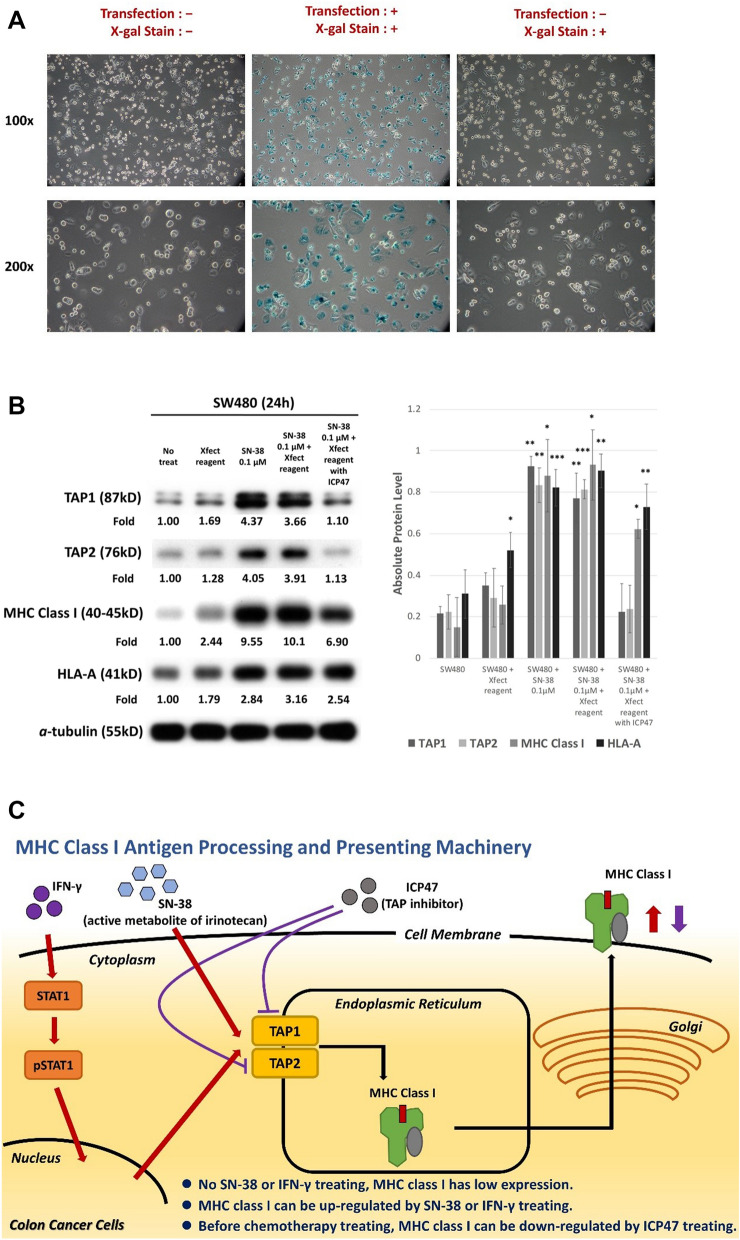
(**A**) Transfection with Xfect plus X-gal into SW480. The blue coloring indicated successful transfection after beta-galactosidase control. (**B**) Western blot for SW480 antigen processing pathway in response to chemotherapy agents with or without ICP47. The full blot was displayed in Supplement figures. Photoshop was applied for only modifying brightness. ImageJ was applied for each signals to quantification. The results of quantification and statistical analysis were shown in the right side of this figures. Quantification of western blot for SW480 antigen processing pathway in response to chemotherapy agents with or without ICP47 by using ImageJ and statistical analysis by using SPSS with at least three or more independent tests. (*P < 0.05, **P < 0.01, ***P < 0.001). (**C**) Mechanism of stimulation for MHC class I expression of chemotherapy agents on colon cancer cells.

 ICP47, which was a herpes simplex virus 1 (HSV-1) product, could specifically inhibit human TAP^32,33^. ICP47 had to be transfected into cells with Xfect. Initially we tested the transfection efficacy through X-gal staining (Fig. 4A). After transfection with ICP47, the stimulation effects of SN-38 on MHC class I and HLA-A were diminished by ICP47, which blocked the upregulation of TAP1 and TAP2 (Fig. 4B). These results confirmed that the stimulation effect on the expression of MHC class I mainly came from the upregulation of TAP1/TAP2 (Fig. 4C).

### SN-38 significantly promoted the phagocytosis of colon cancer cells by monocyte-derived dendritic cells (MoDCs)

**Figure 5:**
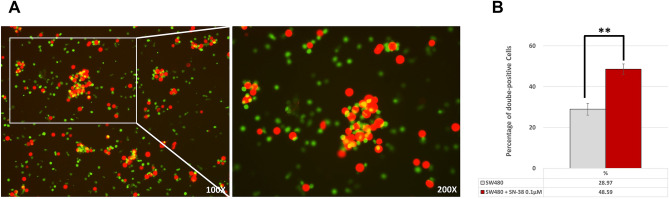
(**A**) Confocal images demonstrated MoDCs (green) and SW480 cells (red). The yellow-colored cells indicated SW480 cells engulfed by MoDCs, i.e. CFSE-DDAO-double-positive cells. (**B**) Percentage of CFSE-DDAO-double-positive cells after co-culture with MoDCs and SW480 with/without SN-38 exposure. (*P < 0.05, **P < 0.01, ***P < 0.001).

 SW480 cells were stained with DDAO (red-colored) and MoDCs were stained with CFSE (green-colored). After co-culture with MoDCs and SW480, CFSE-DDAO-double-positive cells would be observed as yellow-colored If phagocytosis developed by MoDCs (Fig. 5A). As shown on Fig. 5B, the ratio of CFSE-DDAO-double-positive cells significantly increased after SN-38 treatment. This result indicated that the upregulation of MHC class I on SW480 cells induced by SN-38 made the SW480 cells more vulnerable to immune surveillance.

### MHC class I, PD-1, and programmed death ligand 1 (PD-L1) expression increased after first-line chemotherapy and targeted therapy from real-world patient’s samples

**Figure 6:**
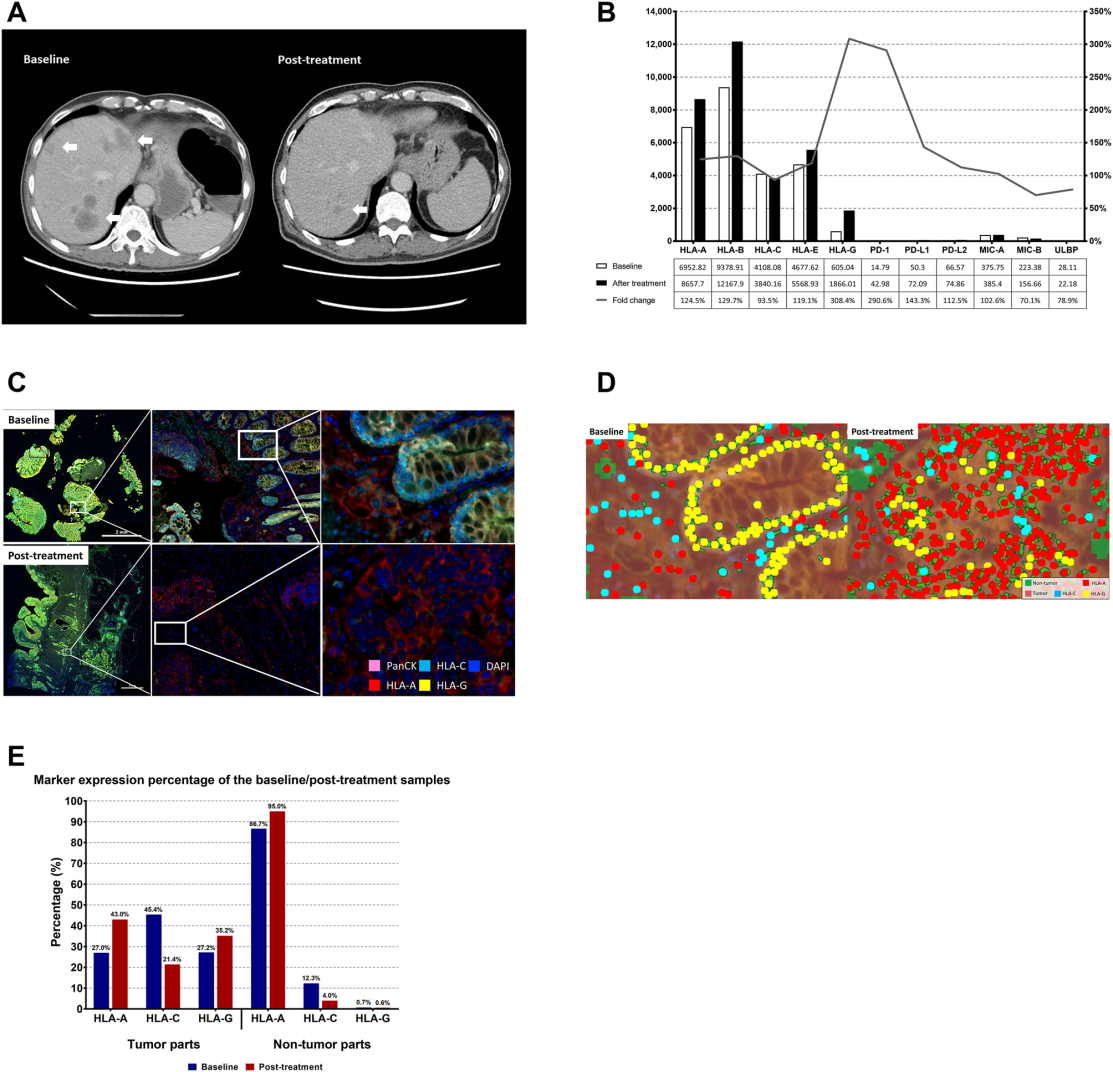
(**A**) CT scan of liver metastases from the patient before (baseline) and after (post-treatment) systemic targeted therapy and chemotherapy. Arrows indicate locations of liver metastases. (**B**) Counts for mRNA of MHC class I, NK cell ligands, PD-1, PD-L1, and PD-L2 from tumors at baseline (white bar) and after treatment (black bar) by NanoString. Grey line indicates the fold change shown as percentage. (**C**) IHC staining from the tumor tissue before (baseline) and after (post-treatment) systemic targeted therapy and chemotherapy. DAPI staining indicated the nucleus. Pan-CK staining indicated tumor cells. All immunofluorescence signals were within one image. (**D**) Computerized scanning of IHC staining from the tumor tissue before (baseline) and after (post-treatment) systemic targeted therapy and chemotherapy. The tumor parts were defined as pan-CK positive and DAPI-positive cells. The nontumor parts were defined as pan-CK negative and DAPI-positive cells. The whole field was then counted with a computer. (**E**) Percentage of marker expression for MHC class I, from tumor at baseline (blue bar) and after treatment (red bar).

 We enrolled patients with de novo mCRC. The 62 year-old man who had stage-IV sigmoid colon cancer initially presented with multiple liver and lung metastases. The expanded RAS and BRAF V600E status were all wild type. The mismatch repair proteins were all preserved. The patient received first-line therapy of cetuximab in combination with irinotecan and infusional 5-FU. Almost complete remission was achieved (Fig. 6A), and the patient thus received laparoscopic low anterior resection for sigmoid tumor and radiofrequency ablation for liver metastases after eight cycles of systemic therapies. We obtained tumor tissues from primary sigmoid tumors before and after treatment and subjected these samples to NanoString analysis. Except for HLA-C, all MHC class I alleles were significantly upregulated (Fig. 6B). MHC class II alleles were also significantly upregulated (Supplementary data). Notably, the expression of programmed death-1 (PD-1), programmed death-1 ligand (PD-L1), and programmed death-2 ligand (PD-L2) also increased after treatment. By contrast, natural killer (NK) cell ligands, including MHC class I-related chains A and B (MIC A, MIC-B) and UL16 binding protein (ULBP), demonstrated no significant change in expression. We reconfirmed these results with the Perkin-Elmer Opal multiplex system of pathological samples, which combined all IHC staining signals within one image. We observed that, comparing the image before and after treatment, the expression of HLA-A significantly increased (Fig. 6C). Using computerized scanning to differentiate tumor parts (both pan-CK positive & DAPI-positive cells) and nontumor parts (pan-CK negative & DAPI-positive cells, Fig. 6D), the results were similar to those from NanoString analysis. The HLA-A were significantly upregulated after treatment both in tumor and nontumor parts. HLA-G were upregulated only in the tumor parts (Fig. 6E).

## Discussion

In our study, we indicated that the expression of MHC class I, PD-1, and PD-L1 significantly increased after first-line chemotherapy and targeted therapy from the samples of real-world patients with de novo mCRC. Using cell line models, we confirmed that chemotherapy agents, particularly SN-38, the active metabolite of irinotecan, stimulated the expression of stimulatory MHC class I alleles through stimulation of the TAP1 and TAP2 pathway expression. Application of ICP47, a specific TAP inhibitor, significantly inhibited expression of TAP1 and TAP2 as well as the expression of downstream MHC class I.

Currently, clinical trials have failed to demonstrate the efficacy of immunotherapy with single ICIs for patients with microsatellite stable (MSS) mCRC. In cohort 2 of the MODUL study, reported at the 2018 European Society for Medical Oncology (ESMO) annual meeting, enrolled patients were randomized into two arms. Patients in the control arm, who received bevacizumab plus 5-FU, had a median overall survival (mOS) of 22.05 months. Patients in the study arm who received bevacizumab plus 5-FU in combination with immunotherapy atezolizumab had mOS of 21.91 months^34^. Although this is a negative study, a long “tail” characterizes the survival curve for patients who received atezolizumab, which usually signifies the efficacy for immunotherapy. Our results indicate that although all three chemotherapy agents stimulated MHC class I expression, SN-38 provided the greatest efficacy, particularly starting from low doses. Chemotherapy agents also stimulated the expression of PD-1 and PD-L1. Our results provided the rationale and a real combination strategy for further immunotherapy for mCRC.

Our study focused on chemotherapy agents for mCRC. The current standard of care for first-line treatment of mCRC is the use of single targeted agents plus doublet chemotherapy regimens^35,36^. Although targeted therapy increased overall response in combination with chemotherapy agents, monotherapy of single targeted agents demonstrated extremely low response rates, which implies that chemotherapy agents exerted greater effects on mCRC treatments. In the KEYNOTE-048 study, pembrolizumab plus platinum and 5-fluorouracil were considered a superior first-line treatment for recurrent or metastatic head and neck cancer compared with the prior standard treatment of cetuximab plus platinum and 5-fluorouracil^37^. This study also implied that immunotherapy in combination with chemotherapy, rather than with targeted therapy, would provide an alternative further clinical trial design.

Our study nonetheless has some limitations: First, our data supported only some but not all mCRC treatments. Metastatic colorectal cancer was so heterogeneous that no single regimen could be applied for all mCRCs. Currently, molecular subtypes of mCRC have been classified into at least four to six categories^38,39^. More combination strategies are required to overcome various molecular subtypes and mCRC immune microenvironment. Second, combination doublet chemotherapy with targeted therapy was a standard of care as mentioned. Our study simplified the treatment agents one by one, but the complexity of interactions among plentiful combination regimens required more research. Third, we also required more clinical samples to confirm and validate details of our hypotheses. Finally, clinical trials to apply these results in routine practice are crucial.

Our study might illuminate the subject of immunotherapy in mCRC treatment and provide new rationale for novel immunotherapy combinations. Chemotherapy agents were the backbone of treatment in the targeted therapy era and continue to be so in the immunotherapy era.

## Methods

### Cell lines

We used a panel of colon cancer cell lines, including SW480, HT29, and COLO320. All cell lines were purchased from the American Type Culture Collection. The cells were planted in T75 culture flasks (Thermo Scientific, 156,499, Waltham, MA, U.S.) and maintained in RPMI-1640 medium (Gibco, 31,800-022, Waltham, MA, U.S.) supplemented with 10% fetal bovine serum (FBS, Gibco, 10,437-028, Waltham, MA, U.S.) and 1% amphotericin B, penicillin, and streptomycin (Antibiotic–Antimycotic, Gibco, 15,240-062, Waltham, MA, U.S.) in an atmosphere of 95% O_2_ and 5% CO_2_ at 37 °C.

### Chemicals and other reagents

Compounds used in this study for colon cancer cell lines were 7-ethyl-10-hydroxycamptothecin (SN-38; Sigma-Merck, H0165, St. Louis, MO, U.S.), fluorouracil (5-FU; Sigma-Merck, F6627, St. Louis, MO, U.S.), oxaliplatin (Sigma-Merck, O9512, St. Louis, MO, U.S.), and interferon-γ (IFN-γ, R&D SYSTEMS, 285-IF-100, Minneapolis, MN, U.S.).

Antibodies used for flow cytometry were phycoerythrin (PE) antihuman pan-MHC class I (BioLegend, 311,406, San Diego, CA, U.S.), purified antihuman human leukocyte antigen-A (HLA-A; My BioSource, MBS438658, Vancouver, BC, Canada), PE anti-mouse immunoglobin G1 (IgG1; BioLegend, 406,608, San Diego, CA, U.S.), purified antihuman human leukocyte antigen-C (HLA-C; BioLegend, 373,302, San Diego, CA, U.S.), PE antimouse IgG2b (IgG2b; BioLegend, 406,708, San Diego, CA, U.S.), PE antihuman human leukocyte antigen-E (HLA-E; BioLegend, 342,604, San Diego, CA, U.S.), PE antihuman human leukocyte antigen-F (HLA-F; BioLegend, 373,204, San Diego, CA, U.S.), PE antihuman human leukocyte antigen-G (HLA-G; BioLegend, 335,906, San Diego, CA, U.S.), PE antihuman MHC class I-related chains A and B (MIC A/B; BioLegend, 320,906, San Diego, CA, U.S.), PE antihuman UL16 binding protein-1 (ULBP-1; R&D SYSTEMS, FAB1380P, Minneapolis, MN, U.S.), PE-IgG1 isotype control (BioLegend, 400,112, San Diego, CA, U.S.), PE IgG2a isotype control (BioLegend, 400,212, San Diego, CA, U.S.), and PE-IgG2b isotype control (BioLegend, 402,204, San Diego, CA, U.S.). The antibodies used for Western blot were signal transducer and activator of transcription 1 (STAT1; CST, #9172, Danvers, MA, U.S), phosphorylated signal transducers and activators of transcription 1 (pSTAT1, CST, #9167, Danvers, MA, U.S.), pan-MHC class I (Origene, AM33035PU-N, Rockville, MD), transporter of antigen processing 1 (TAP1; Abcam, ab83817, Cambridge, UK), transporter of antigen processing 2 (TAP2; Abcam, ab180611, Cambridge, UK), tapasin (Abcam, ab13518, Cambridge, UK), endoplasmic reticulum-resident protein 57 (ERp57; Abcam, ab10287, Cambridge, UK), endoplasmic reticulum aminopeptidase 1 (ERAP1; Abcam, ab124669, Cambridge, UK), endoplasmic reticulum aminopeptidase 2 (ERAP2; Abcam, ab69037, Cambridge, UK), Calreticulin (Abcam, ab2907, Cambridge, UK), Calnexin (Abcam, ab10286, Cambridge, UK), HLA-A (Abcam, ab52922, Cambridge, UK), HLA-C (Abcam, ab193432, Cambridge, UK), ß-actin (Abcam, ab8227, Cambridge, UK), α-tubulin (Millipore, #05-829, Burlington, MA, U.S.), horseradish peroxidase (HRP) donkey anti-rabbit IgG (BioLegend, 406,401, San Diego, CA, US), and HRP goat anti-mouse IgG (BioLegend, 405,306, San Diego, CA, U.S). The transporter of the antigen processing (TAP) inhibitor and protein transfection reagent used for cell culture were recombinant human herpesvirus 1 infected cell protein 47 (ICP47; US12, MyBioSource, MBS1252493, Vancouver, BC, Canada) and Xfect protein transfection reagent kit (Takara Bio, 631,324, Mountain View, CA, U.S.).

Reagents and cytokines used for isolation of human peripheral blood mononuclear cells (PBMCs) and induction of monocytes-derived dendritic cells (MoDCs) were Ficoll-Paque Plus (GE Healthcare Life Sciences, 17-1440-02, Chicago, IL, U.S.), human CD14^+^ magnetic MicroBeads (Miltenyi Biotec, 130-050-201, Bergisch Gladbach, NRW, Germany), Recombinant Human IL-4 (interleukin-4, PeproTech, 200-04, Cranbury, NJ, U.S.), Recombinant Human GM-CSF (granulocyte–macrophage colony-stimulating factor, PeproTech, 300-03, Cranbury, NJ, U.S.), DDAO (CellTrace Far Red Cell Proliferation Kit, Invitrogen, C34572, Waltham, MA, U.S.), CFSE (CellTrace CFSE Cell Proliferation Kit, Invitrogen, C34570, Waltham, MA, U.S.), IgG from human serum (hIgG, Sigma-Aldrich, I4506, St. Louis, MO, U.S.).

Chemicals used for immunohistochemical (IHC) staining were xylene (J.T. Baker, JT-9490-03, U.S.), ethanol (Sigma-Aldrich, 32,221, Germany), formaldehyde solution (10% in aqueous phosphate buffer) (Macron, H121-08, U.S.), 10 × Tris-buffered saline (TBS) pH 7.4 (Protech, BF204, R.O.C.), TWEEN 20 (MyBioSource, MBS4156394, U.S.), and ProLong Diamond Antifade Mountant (Invitrogen, P36965, U.S.). Antibodies used for IHC staining were HLA-A (Abcam, ab52922, Cambridge, UK), HLA-C (Abcam, ab193432, Cambridge, UK), HLA-G (Abcam, ab52455, Cambridge, UK), and pan Cytokeratin (pan-CK; Abcam, ab7753, Cambridge, UK). The kit used for multiplex IHC staining was the Opal 7 Solid Tumor Immunology Kit (PerkinElmer, OP7TL4001KT, U.S.).

### Flow cytometry

We seeded 10^6^ cells with 10-mL culture medium in 10-cm dishes. The next day, these cells were treated with SN-38, 5-FU, oxaliplatin, or IFN-γ for the subsequent 48 h. The interferon-γ was applied as positive control^40,41^. The cells were then harvested with trypsin (Gibco, 15,400-054, Waltham, MA, U.S.) and centrifuged (500 g, 5 min). In each eppendorf tube, 5 × 10^5^ cells were added into 200 μL of cold phosphate-buffered saline (PBS) and recentrifuged (500 g, 5 min). After removing the supernatant, we added 5 μL of fluorescent antibody (including isotype control antibodies) with 50 μL of cold PBS (antibody:cold PBS = 1:10), placing the sample on ice and in darkness for 20 min. Four hundred microliters of PBS were then added, and centrifugation was performed at 500 g for 5 min twice. The cells were analyzed using a flow cytometer (BD FACS Calibur).

### Western blot

Cells were scraped and washed three times in cold PBS and then resuspended in radioimmunoprecipitation assay lysis, extraction buffer (Thermo Scientific, 89,900, Waltham, MA, U.S.), 1% protease, and 1% phosphatase inhibitor (Thermo Scientific, 78,440, Waltham, MA, U.S.) for 15 min. Centrifugation at 15,000 rpm was then applied for 15 min at 4 °C. Protein concentrations were measured using the Bradford method, electrophoresed (80 V, 130 min) in 10% SDS-PAGE (15 µg per lane), and finally transferred (400 mA, 70 min) onto polyvinylidene fluoride blotting membranes (GE Healthcare Life Sciences, 10,600,023, Chicago, IL, U.S.). For blockage of nonspecific binding sites, the membranes were incubated in 5% nonfat milk in PBS containing 0.2% Tween 20 (MyBioSource, MBS4156394, Vancouver, BC, Canada) for 70 min, and the membranes were incubated overnight at 4 °C with primary antibodies. The membranes were washed with TBS containing 0.2% Tween 20 for 15 min (3 times) and incubated with HRP-conjugated secondary antibodies at room temperature for 1 h and then washed with TBS containing 0.2% Tween 20 for 25 min (3 times). After washing, membranes were incubated with enhanced chemiluminescence Western blotting detection reagents (Millipore, P90720, Burlington, MA, U.S.) and exposed to film for 1 to 10 min. The interferon-γ was applied as positive control^40,41^.

### Protein transfection

We first seeded 4 × 10^5^ cells with 3 mL of culture on a 6-well plate. The next day, TAP-inhibitor ICP47 (5 μg) was added to the tube containing the Xfect protein transfection reagent and incubated at room temperature for 30 min. The mixture was then applied to cells with 400 μL of serum-free medium (RPMI-1640 only) in each well and incubated at 37 °C for 60 min. We added 3 mL of the culture medium to each well and performed incubation at 37 °C for another 2 h. SN-38 (0.1 μM) was treated for 24 or 48 h. As a control, some other cells were stained with X-gal to determine the transfection efficiency through beta-galactosidase control (Takara Bio, 631,326, Mountain View, CA, U.S.).

### Isolation of PBMCs

PBMCs were isolated from peripheral blood of the health human volunteers. We applied Ficoll-Paque Plus and diluted with PBS by density gradient centrifugation (400 g, 20 min, without break). Then, the monocytes were purified by positive selection with human CD14^+^ magnetic MicroBeads by manual MACS cell separation system (Miltenyi Biotec). The monocytes were incubated for 6 days into RPMI-1640 and supplemented with 10% FBS, 1% Antibiotic–Antimycotic, 50 ng/mL GM-CSF and 20 ng/mL IL-4 in an atmosphere of 95% O_2_ and 5% CO_2_ at 37 °C to harvest MoDCs. The induction medium would be renewed at the third day.

### MoDCs-based phagocytosis

After 6 days of induction, MoDCs were stained with 2.5 μM of CFSE (20 min and incubated at 37 °C), and incubated with an excess of hIgG (20 μg/1 × 10^6^ cells, 30 min at room temperature). SW480 were stained with 2.5 μM of DDAO (20 min and incubated at 37 °C), and seeded in 10-cm dishes (1 × 10^6^ cells per dish). The next day, SW480 were treated with SN-38 (0.1 μM) for subsequent 24 h. After totally preparation, MoDCs and SW480 were co-cultured in 1:1 ratio for 2 h. After co-culture, all cells were recovered and analyzed by flow cytometer (BD FACS Calibur). SW480 engulfed by MoDCs would presented co-expression of CFSE and DDAO (double-positive).

### Patient enrollment

We enrolled one patient who had been treated at National Taiwan University Hospital (NTUH). Primary sigmoid tumor tissues were obtained at diagnosis and after curative surgery. We obtained complete medical records including all treatment flows of systemic therapies and regular computed tomography (CT) scan follow-up reports at NTUH. This study was approved by the Institutional Review Board of NTUH (NTUH#201612146RINB), and informed consent was obtained from the patient. All methods were carried out in accordance with relevant guidelines and regulations.

### NanoString assay of patient samples

The NanoString nCounter PanCancer Immune Profiling Panel (NanoString Technologies) is a commercialized multiplexed gene expression panel, which can simultaneously quantitate 770 immune-related genes. This was used for further mRNA quantification of two 150-ng ribonucleic acid extracted from patient’s both tissue samples. All procedures, including preparation, hybridization, detection, scanning, and normalization, were performed according to the manufacturer’s instructions. Detailed information and the gene list are available on the official website at https://www.nanostring.com/products/gene-expression-panels/gene-expression-panels-overview/hallmarks-cancer-gene-expression-panel-collection/pancancer-immune-profiling-panel.

### Immunohistochemical (IHC) staining of patient samples

We applied the Perkin-Elmer Opal multiplex system to simultaneously detect multiple biomarkers plus nuclear counterstain within a single image. Initially, the 5-μm FFPE pathology slides were incubated at 70 °C for 1.5 h, deparaffinized with xylene, and then hydrated through an ethanol gradient ending with distilled water wash. The slides were fixed using 10% neutral buffered formalin for 20 min. Antigen retrieval was subsequently performed with a microwave for 15 min in AR6 (PerkinElmer, AR600250ML, U.S.) or AR9 solution (PerkinElmer, AR900250ML, U.S.), and the slides were rinsed with 0.05% tris-buffered saline–polysorbate 20 buffer and incubated with Antibody Diluent (PerkinElmer, ARD1001EA, U.S.) for 10 min. After the blocking solution was removed, the slides were incubated with the primary antibody for 30 min at room temperature or overnight at 4 °C. The slides were sequentially incubated with Opal Polymer HRP secondary antibody solution (PerkinElmer, ARH1001EA, U.S.) and Opal fluorophore solution (PerkinElmer, FP1487001KT, U.S.) for 10 min at room temperature, respectively. Finally, the slides were incubated with 4′,6-diamidino-2-phenylindole (DAPI) solution (PerkinElmer, FP1490A, U.S.) for 5 min and mounted with ProLong Diamond Antifade Mountant (Invitrogen, P36965, U.S.). Imaging and quantifying of biomarkers in the tissue sections were performed using Vectra Polaris Automated Quantitative Pathology Imaging System along with inForm analysis software (PerkinElmer, CLS143455, U.S.). Detailed information is available on the official website at https://www.perkinelmer.com/lab-solutions/resources/docs/DTS_1-05-40-NR-OPALGUIDELINES_Opal4-7-color_Manual_Kit_Insert.pdf.

### Statistical analysis

All results were collected with at least three or more independent tests. The testing results were presented in average and standard deviations were also demonstrated within figures. We also applied the ImageJ for quantification of western blot (by National Institute of Health, USA). We also performed the paired samples t test or ANOVA test as indicated. All *P*-values were two-tailed and value < 0.05 were considered statistically significant. All data analyses were performed using SPSS version 20.0 software (Chicago, IL, USA).

## Supplementary Information

Supplementary Information
