# Supplementary material for: Chemotherapy agents stimulate dendritic cells against human colon cancer cells through upregulation of the transporter associated with antigen processing
Source: Sci Rep. 2021 Apr 27;11:9080. doi: 10.1038/s41598-021-88648-z (PMC8079421; doi:10.1038/s41598-021-88648-z)
Supplement: Supplementary file 1 — Supplementary Information [file 41598_2021_88648_MOESM1_ESM.pdf]

Chemotherapy agents stimulate dendritic cells against human colon cancer cells through upregulation of the transporter associated with antigen processing

Yi-Hsin Liang[1,3,4,6], Jia-Huei Tsai[5], Yung-Ming Cheng[5], Kuang-Yu Chan[1], Wen-Ling Hsu[3], Chang-Cheng Lee[1,2], Kuo-Hsing Chen[1,4,6], and Kun-Huei Yeh[1,2,4,6]

[1]Graduate Institute of Oncology and [2]Clinical Medicine, [3]Centers of Genomic and Precision Medicine, College of Medicine, National Taiwan University, Taipei, Taiwan, R.O.C;

Departments of [4]Oncology, Department of [5]Pathology, [6]National Taiwan University Cancer Center, National Taiwan University Hospital, Taipei, Taiwan, R.O.C;

Correspondence to: Kun-Huei Yeh, MD, Ph.D., Professor and Director of Department of Oncology, National Taiwan University Hospital, No 7, Chung-Shan South Rd, Taipei 10002, Taiwan, R.O.C. Tel.: +886223123456, ext. 67514, Fax: +886223711174, e-mail: khyeh@ntu.edu.tw

COLO320

IFN-γ : 100U/mL, 48h
MFI : mean fluorescence intensity

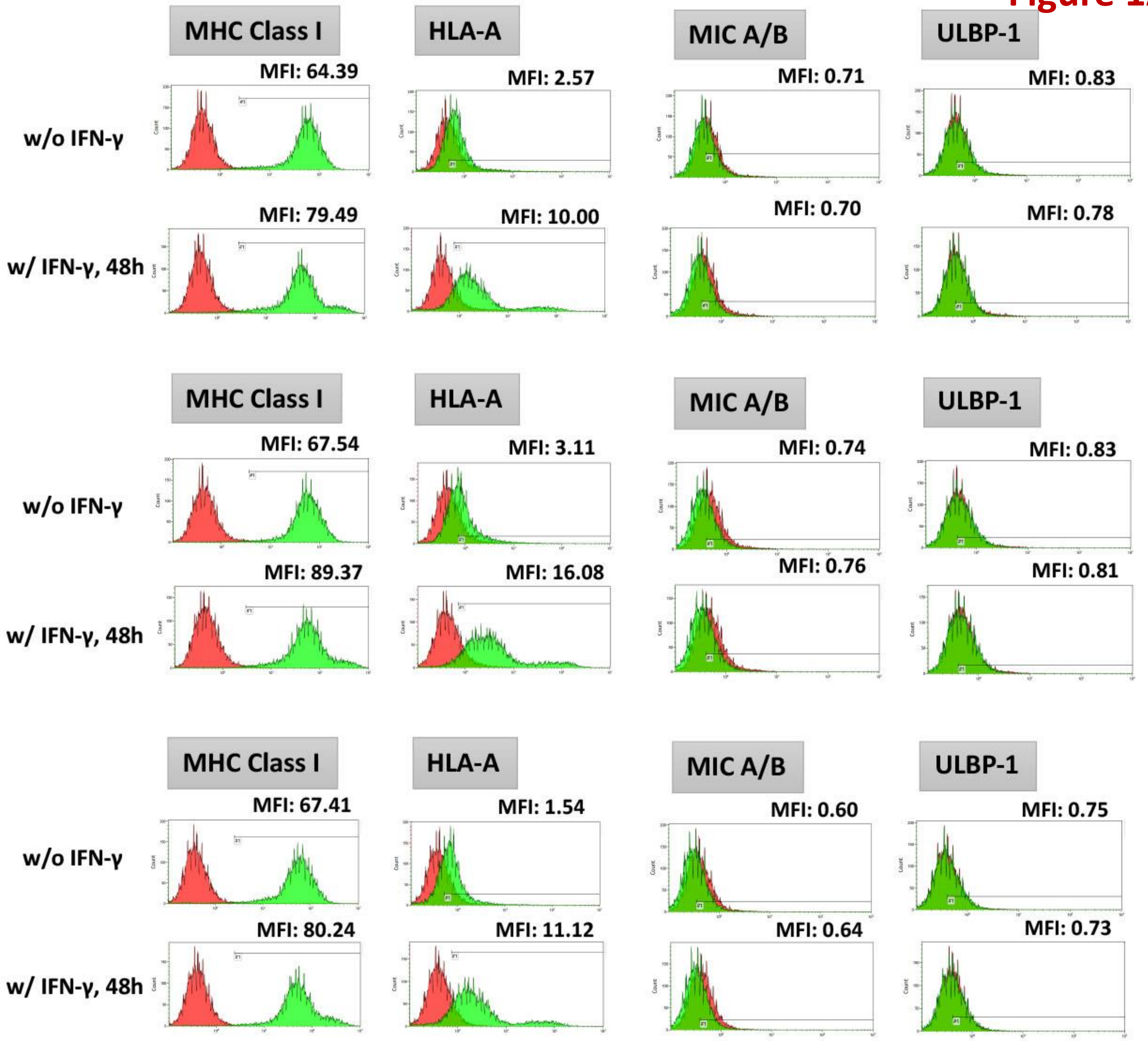

Figure 1A, 1C

HT29

IFN-γ : 100U/mL, 48h

MFI : mean fluorescence intensity

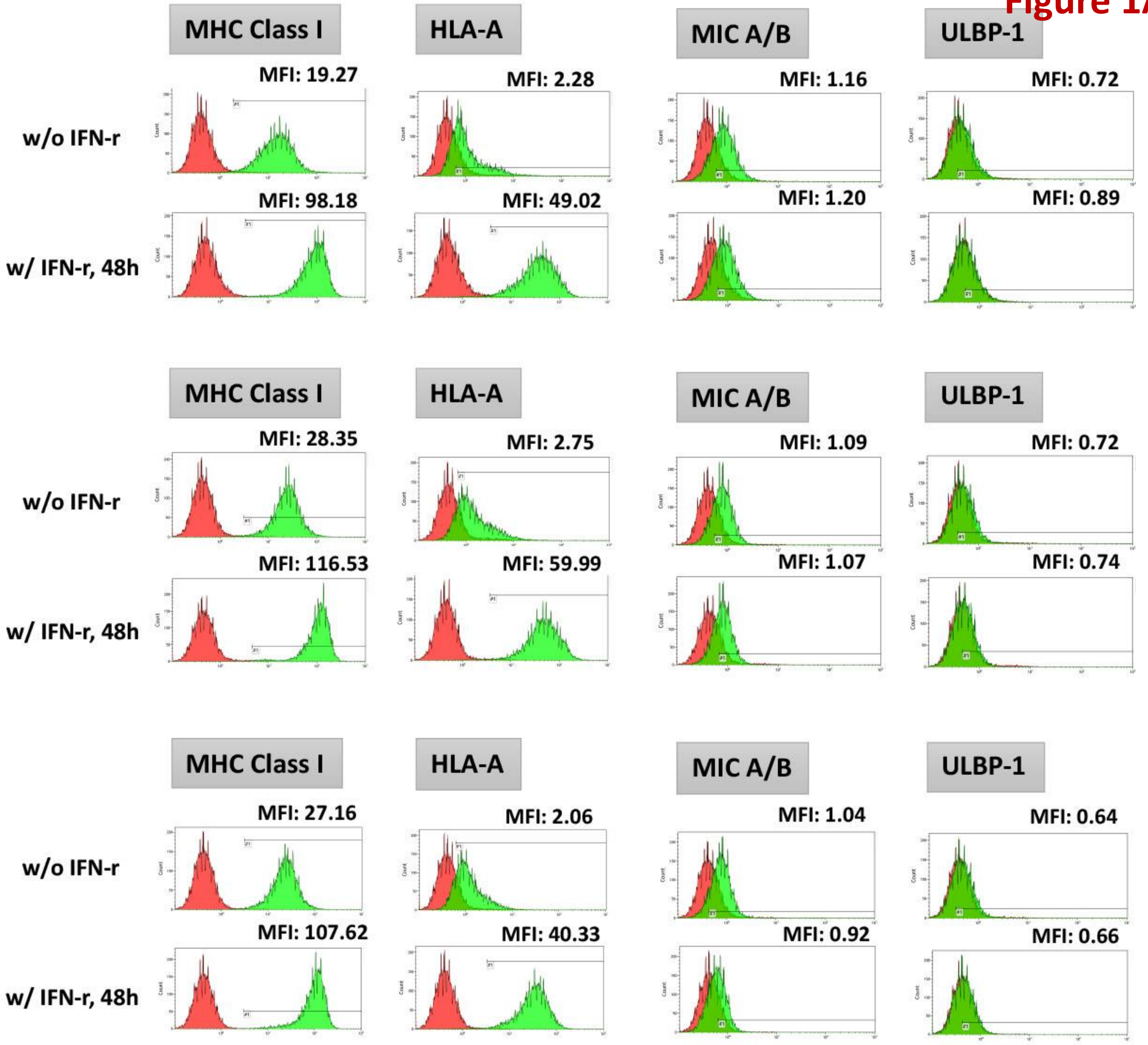

Figure 1B, 1C

SW480

IFN-γ : 100U/mL, 48h

MFI : mean fluorescence intensity

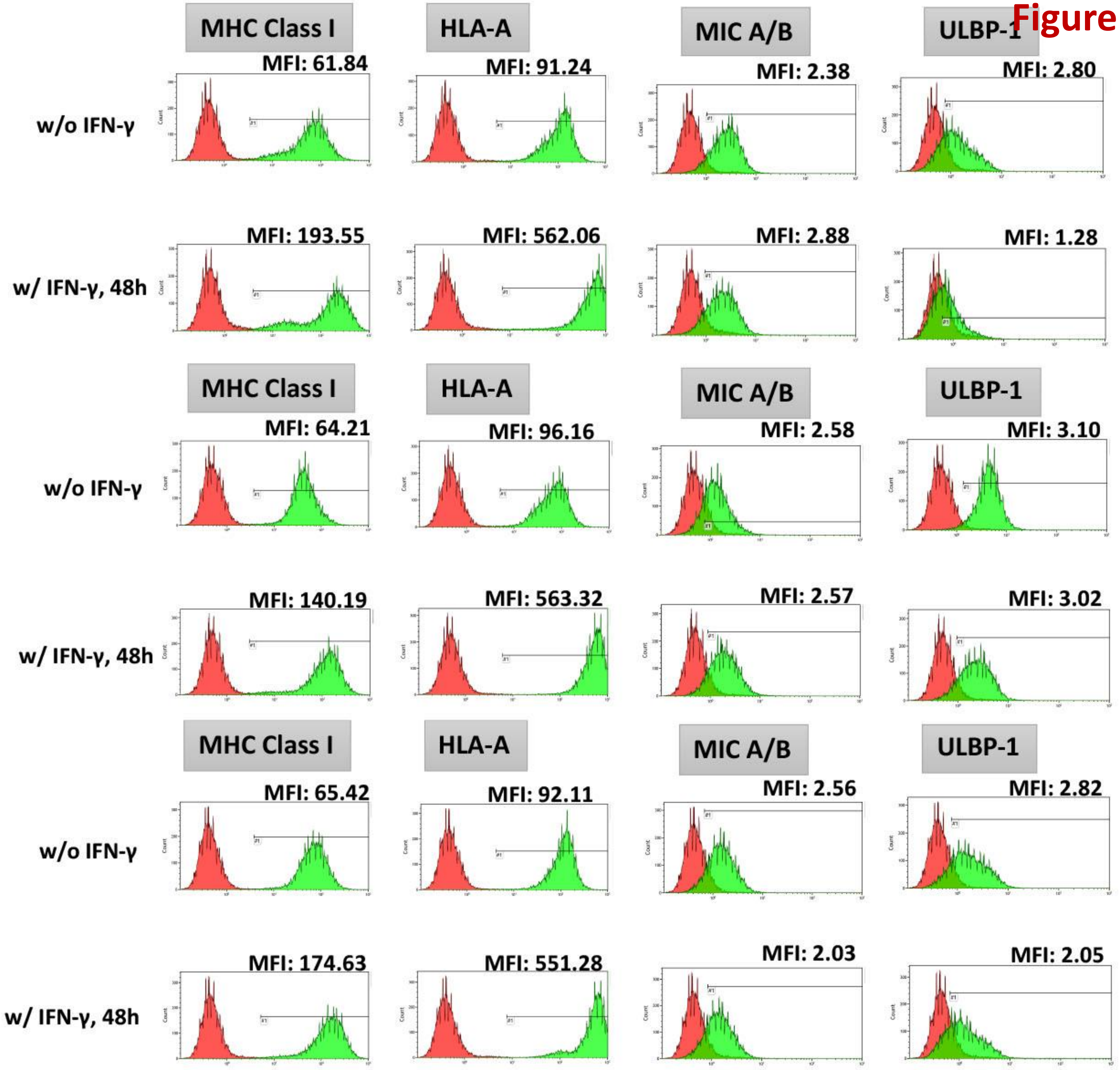

Figure 1D

SW480

IFN-γ : 100U/mL, 48h

MFI : mean fluorescence intensity

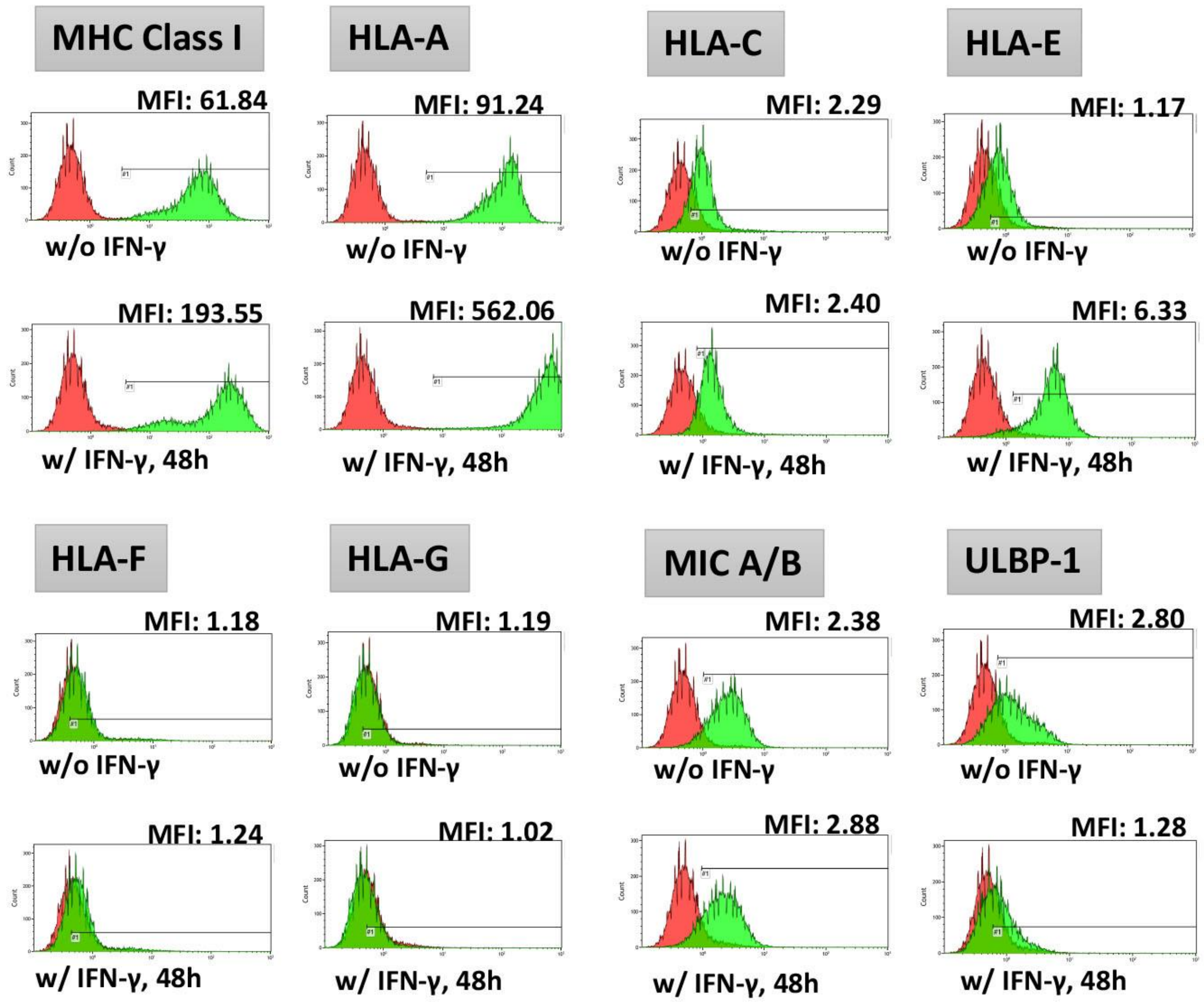

Figure 1D

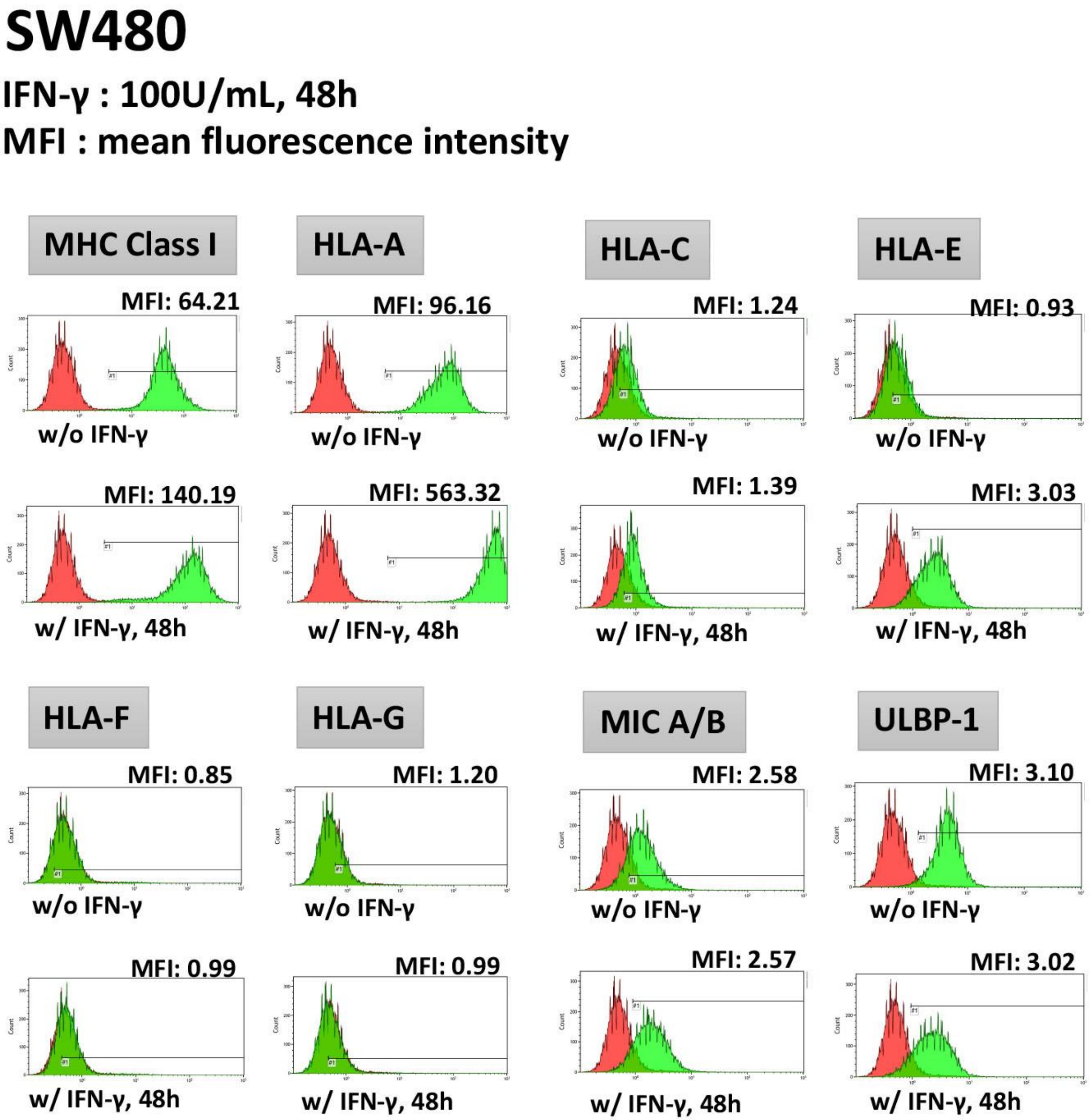
SW480
IFN-γ : 100U/mL, 48h
MFI : mean fluorescence intensity
MHC Class I
MFI: 64.21
w/o IFN-γ
MFI: 140.19
w/ IFN-γ, 48h
HLA-A
MFI: 96.16
w/o IFN-γ
MFI: 563.32
w/ IFN-γ, 48h
HLA-C
MFI: 1.24
w/o IFN-γ
MFI: 1.39
w/ IFN-γ, 48h
HLA-E
MFI: 0.93
w/o IFN-γ
MFI: 3.03
w/ IFN-γ, 48h
HLA-F
MFI: 0.85
w/o IFN-γ
MFI: 0.99
w/ IFN-γ, 48h
HLA-G
MFI: 1.20
w/o IFN-γ
MFI: 0.99
w/ IFN-γ, 48h
MIC A/B
MFI: 2.58
w/o IFN-γ
MFI: 2.57
w/ IFN-γ, 48h
ULBP-1
MFI: 3.10
w/o IFN-γ
MFI: 3.02
w/ IFN-γ, 48h

Figure 1D

SW480

IFN-γ : 100U/mL, 48h

MFI : mean fluorescence intensity

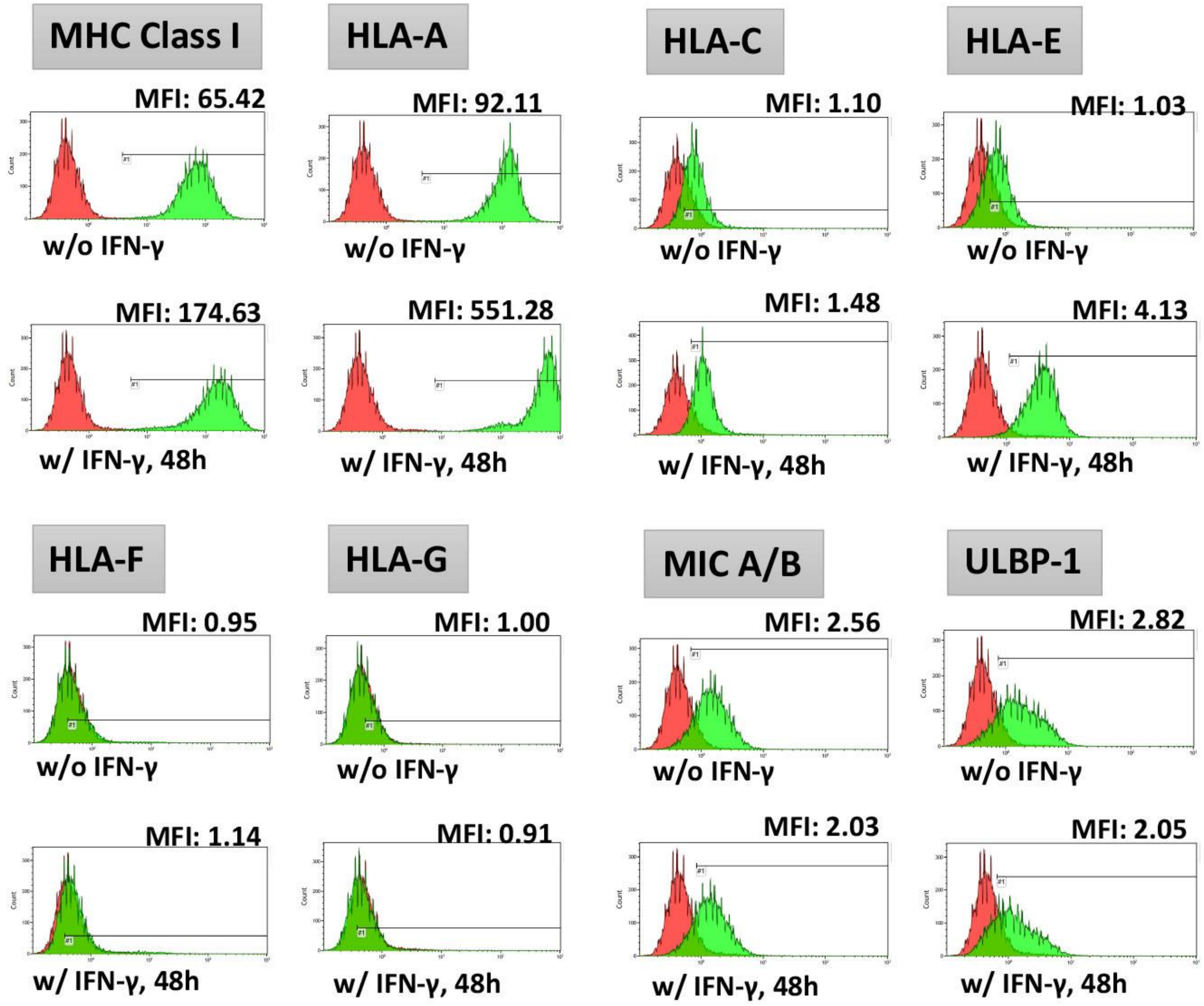

Figure 2A, 2B

SW480

MFI : mean fluorescence intensity

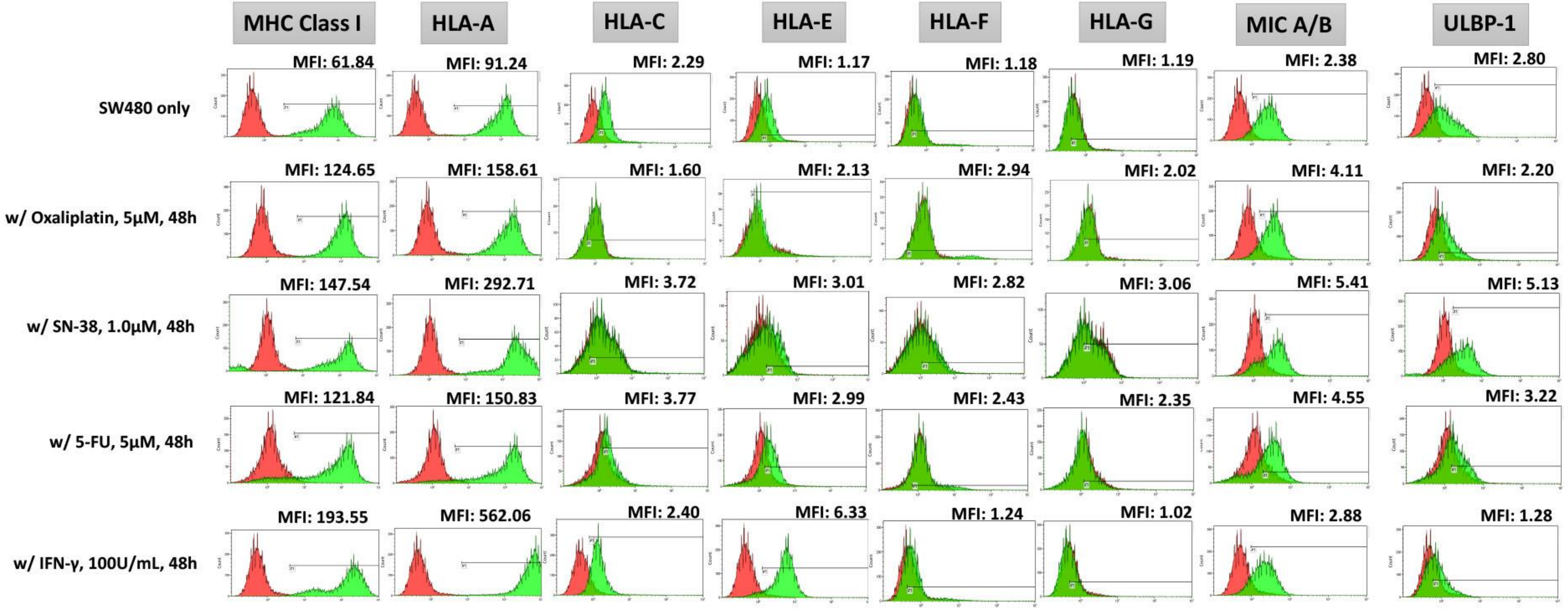

Figure 2A, 2B

SW480

MFI : mean fluorescence intensity

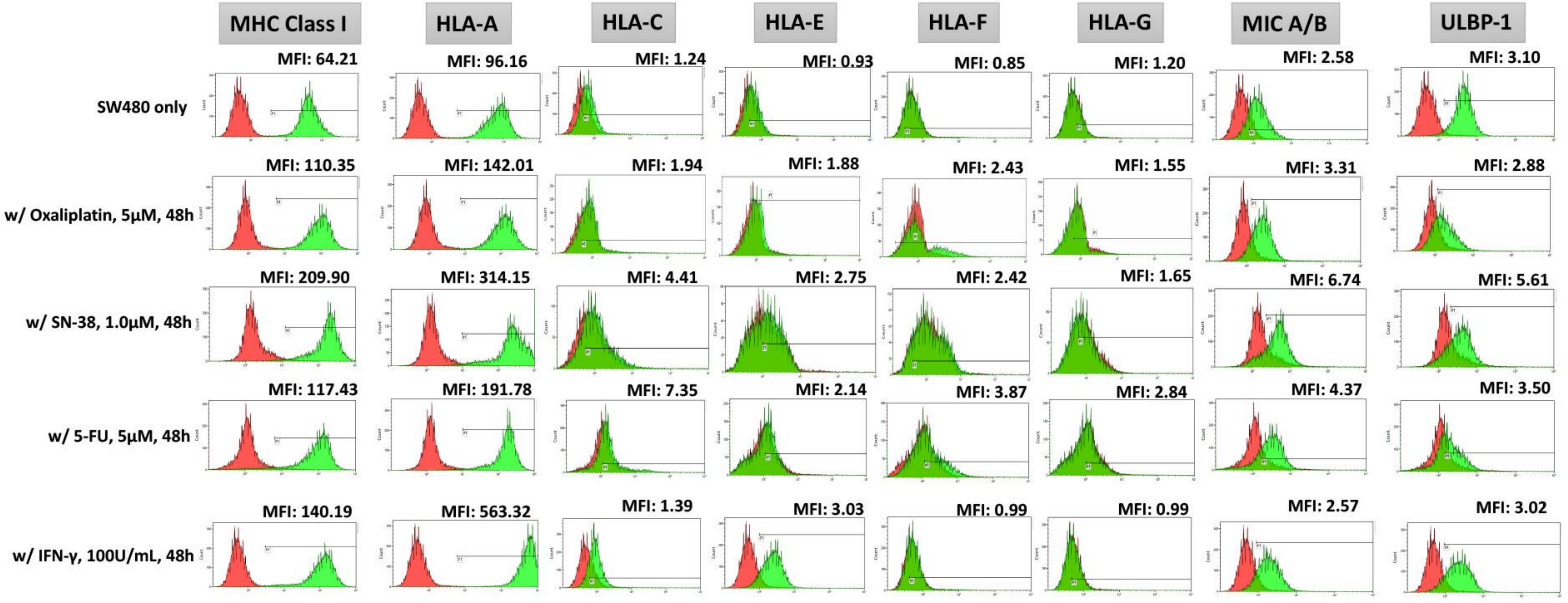

Figure 2A, 2B

SW480

MFI : mean fluorescence intensity

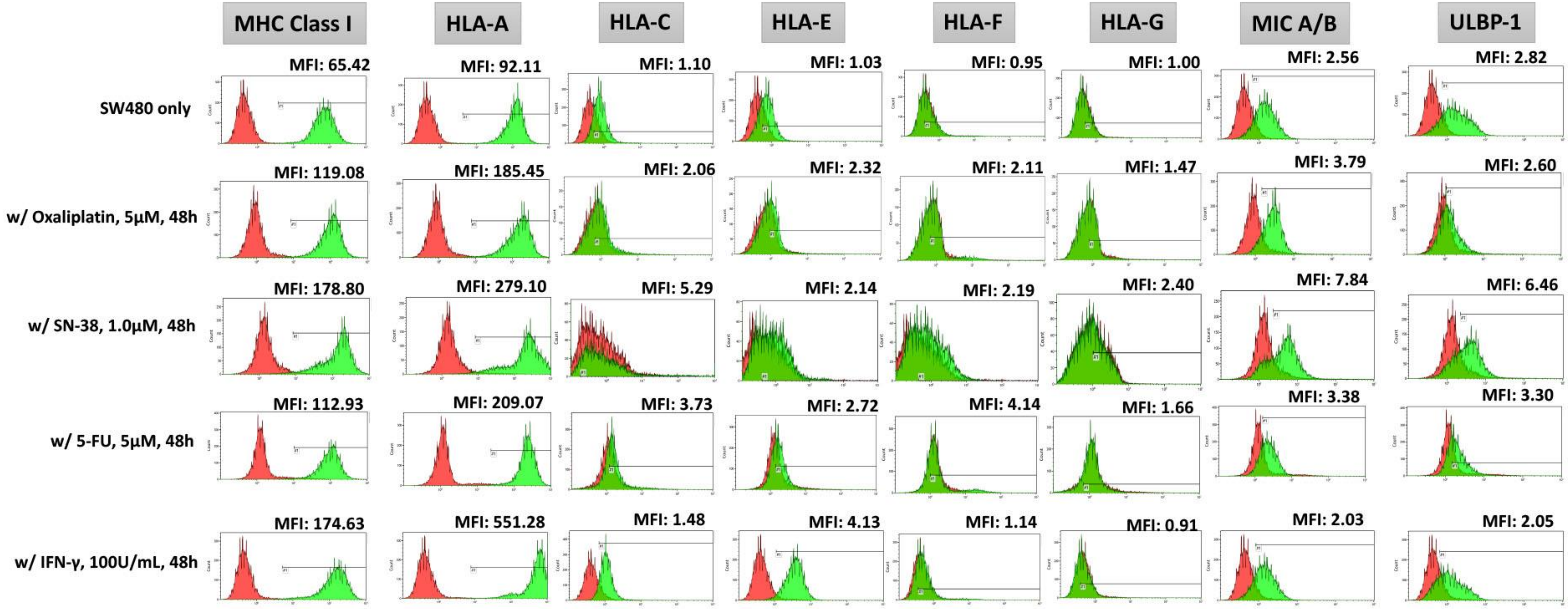

Figure 2C, 2D

SW480

SN-38, 48h

MFI : mean fluorescence intensity

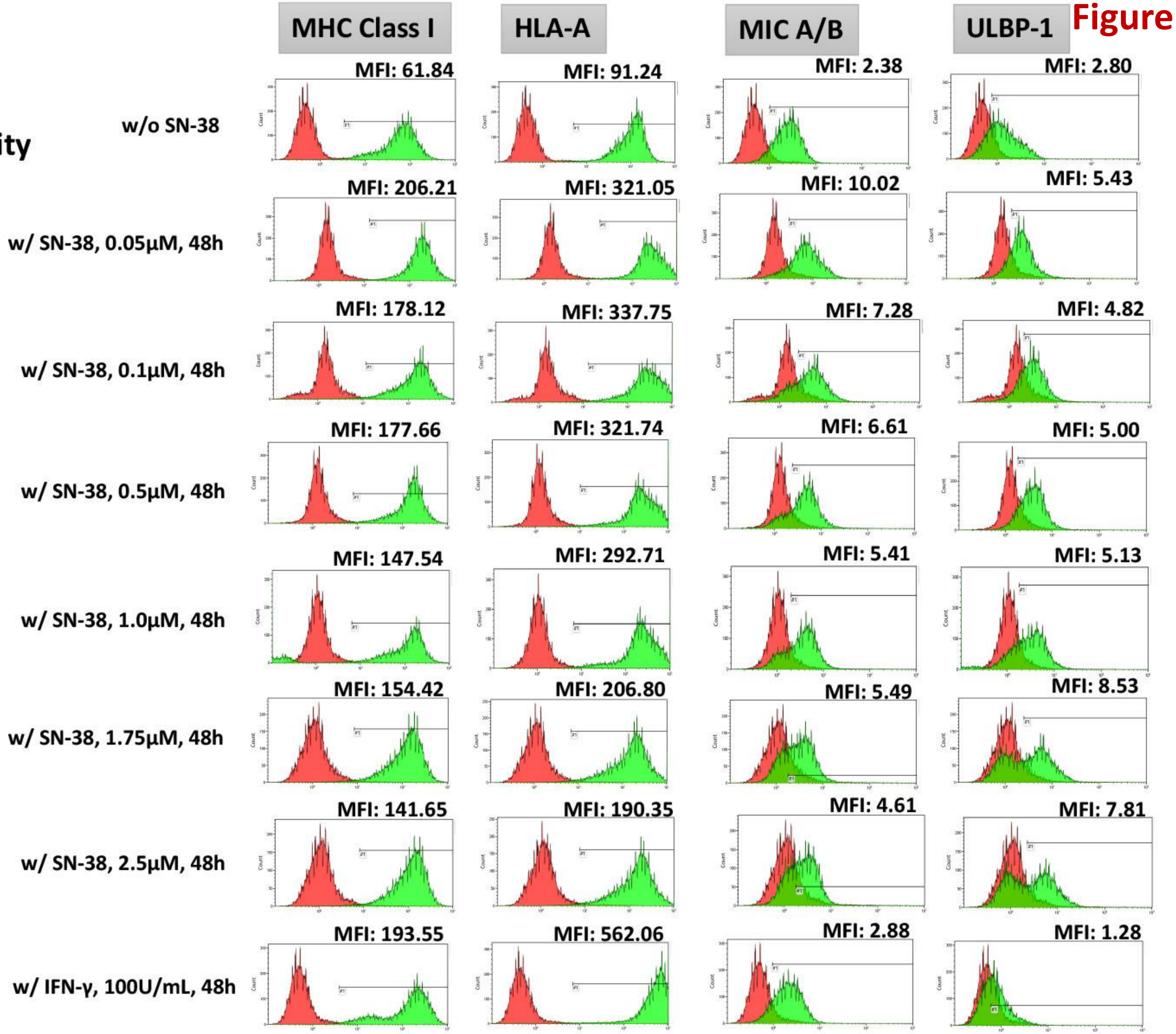

Figure 2C, 2D

SW480

SN-38, 48h

MFI : mean fluorescence intensity

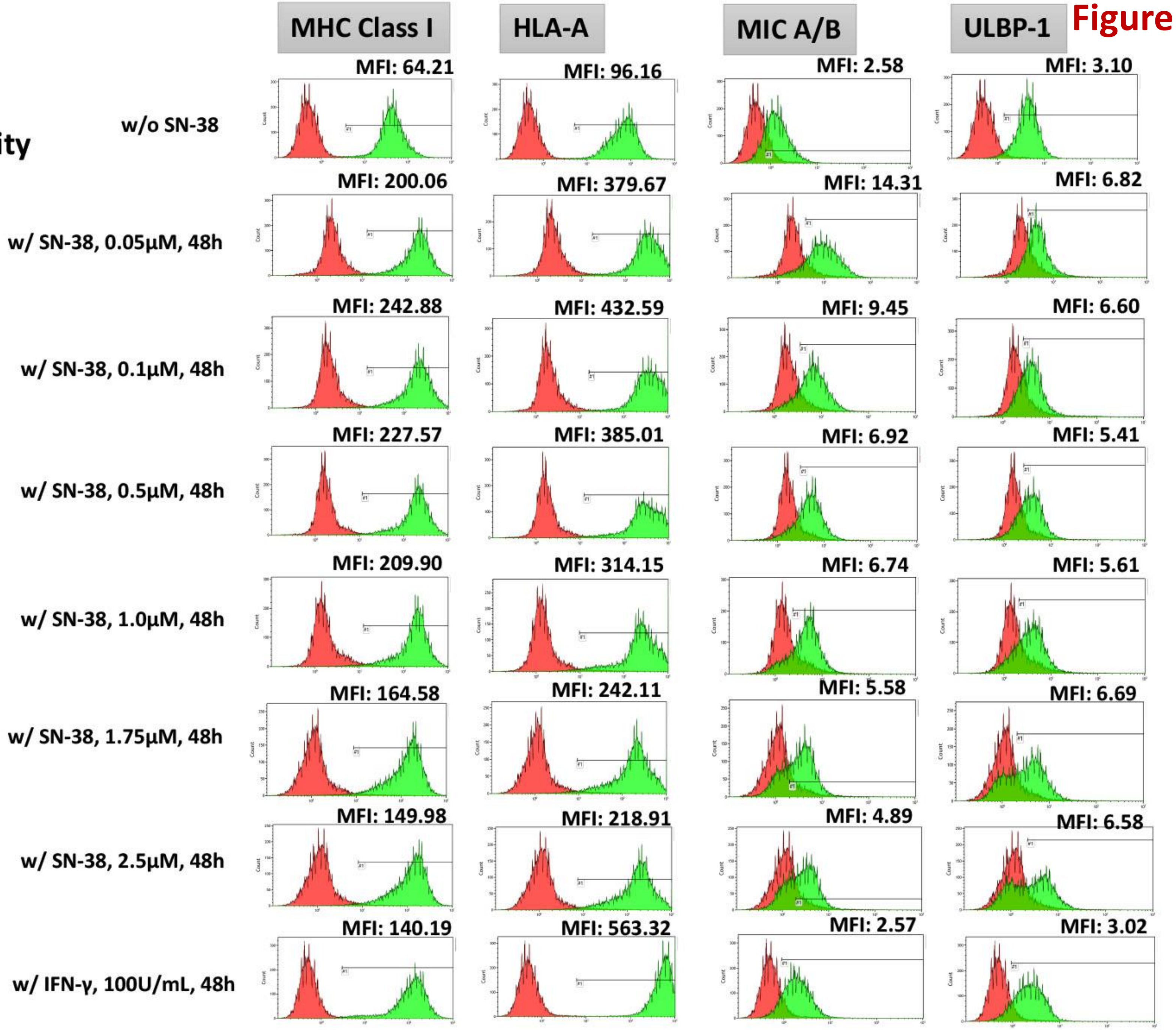

Figure 2C, 2D

SW480

SN-38, 48h

MFI : mean fluorescence intensity

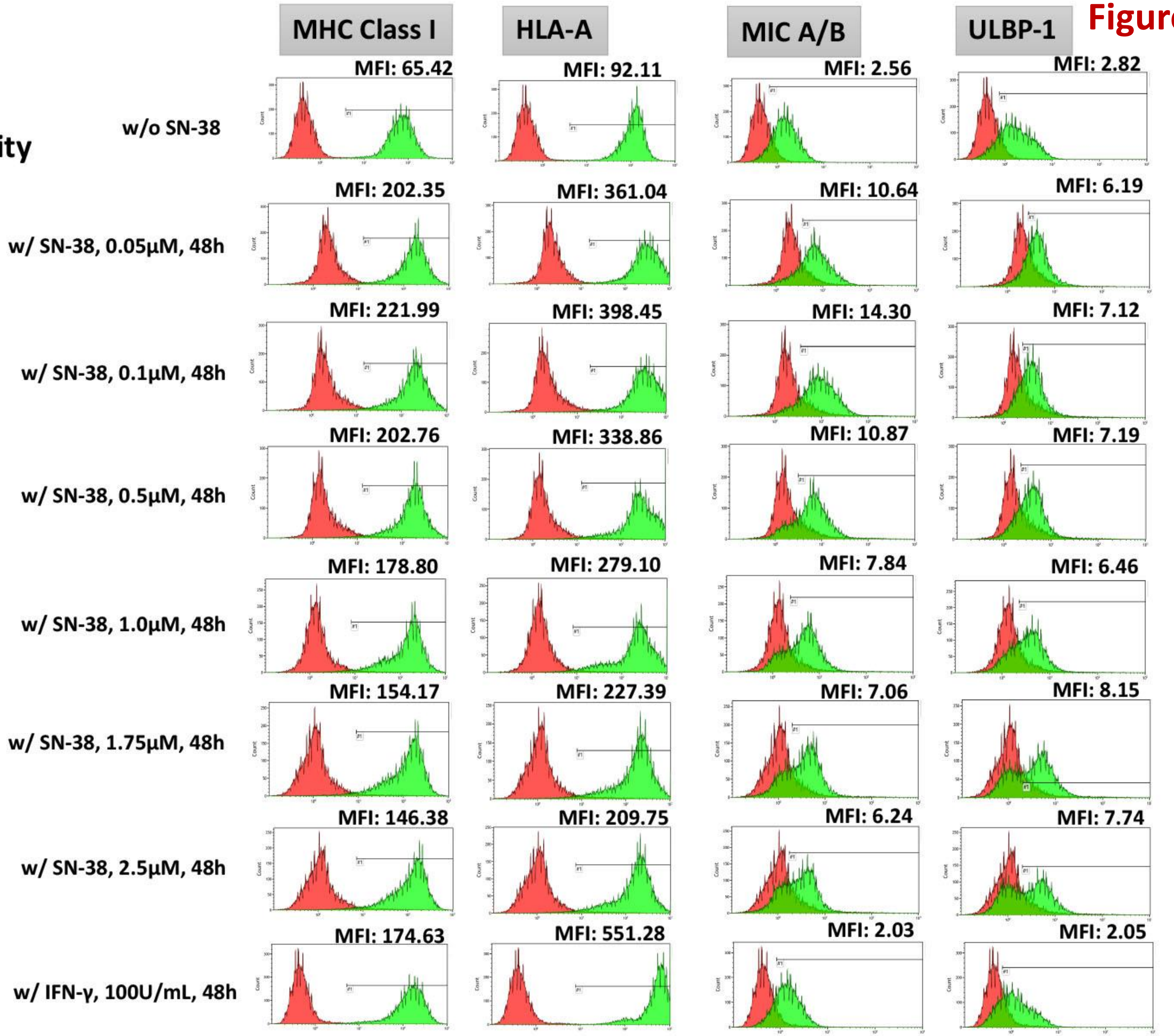

Figure 2E, 2F

SW480

Oxaliplatin, 48h

MFI : mean fluorescence intensity

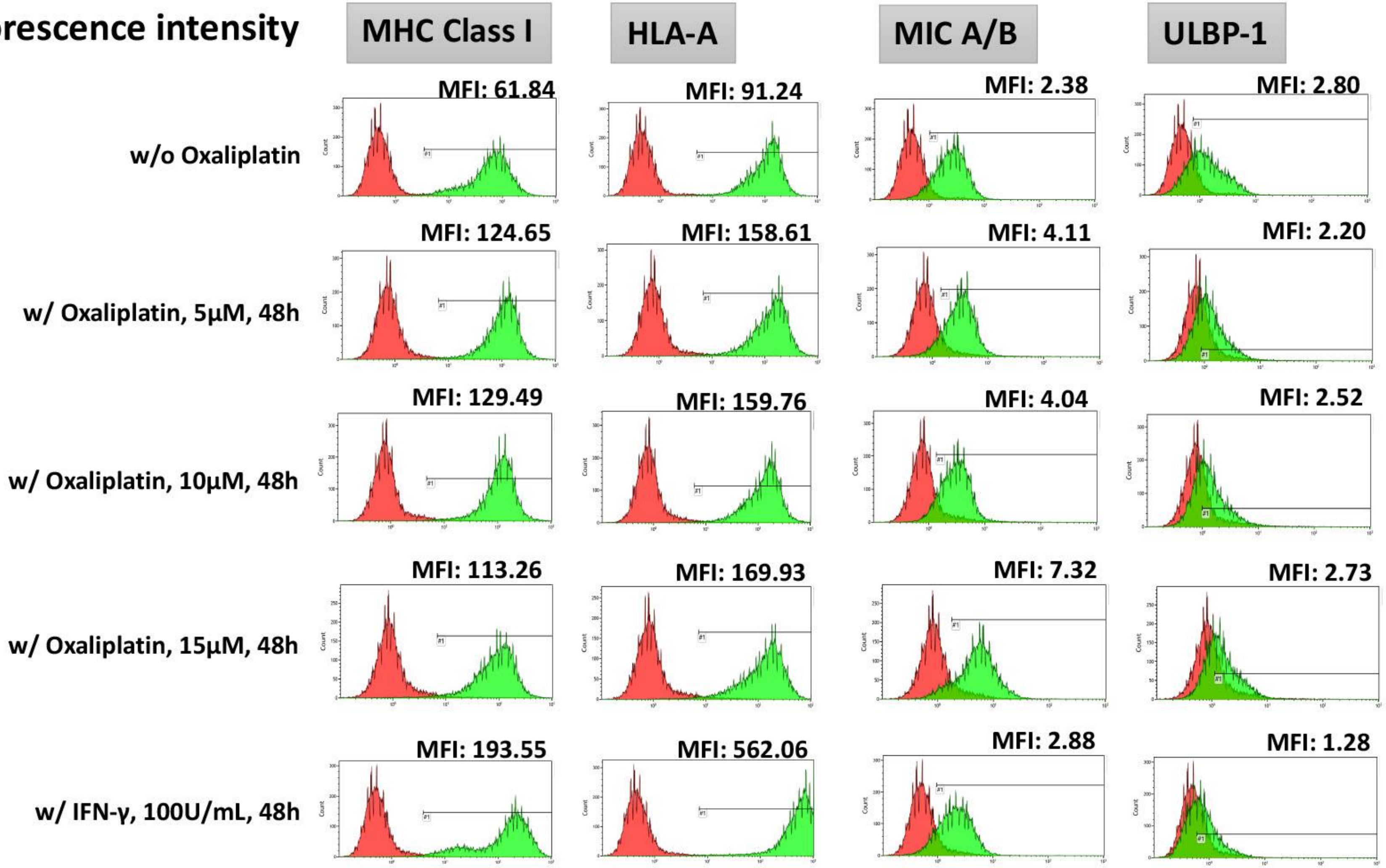

Figure 2E, 2F

SW480

Oxaliplatin, 48h
MFI : mean fluorescence intensity

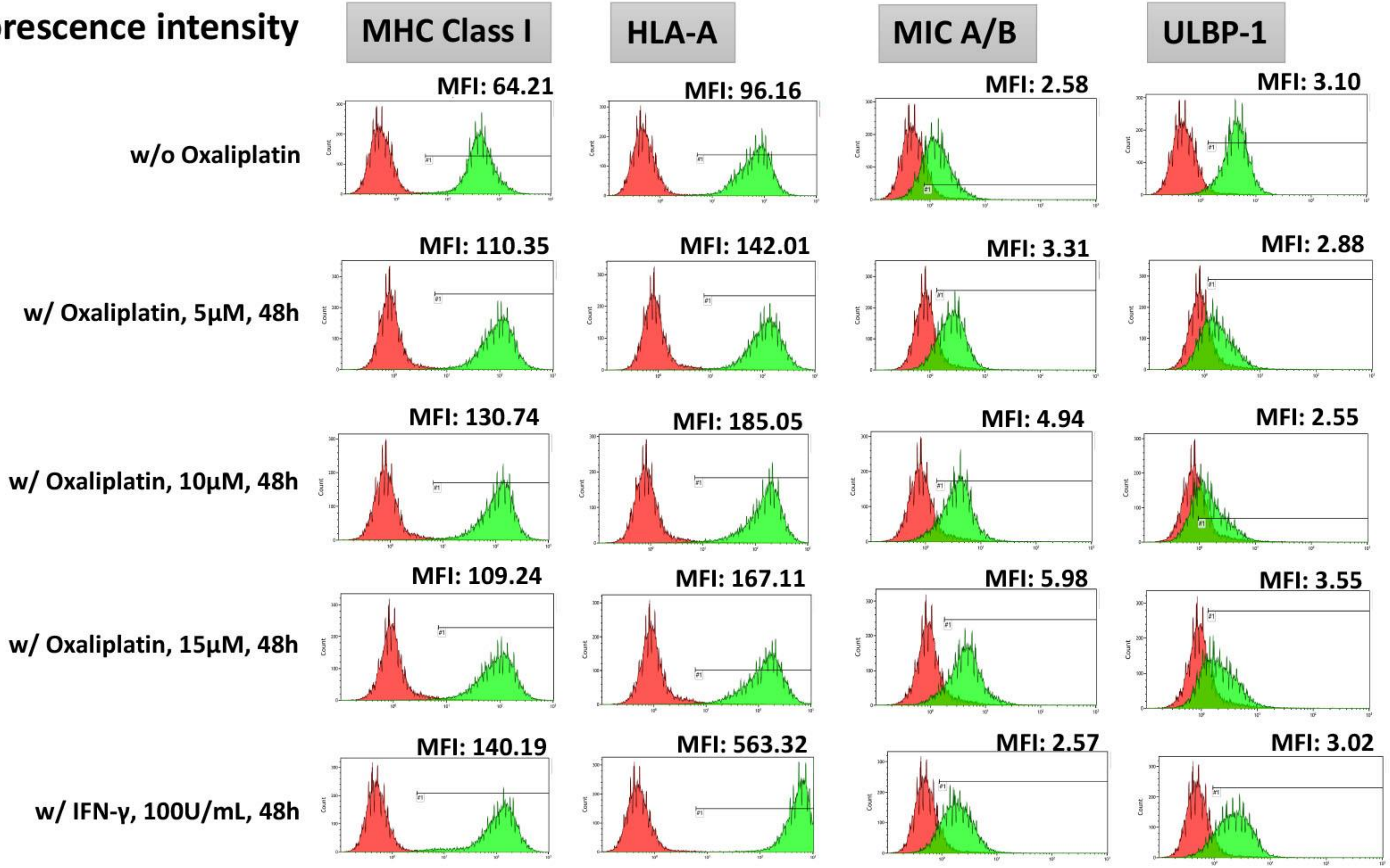

Figure 2E, 2F

SW480

Oxaliplatin, 48h

MFI : mean fluorescence intensity

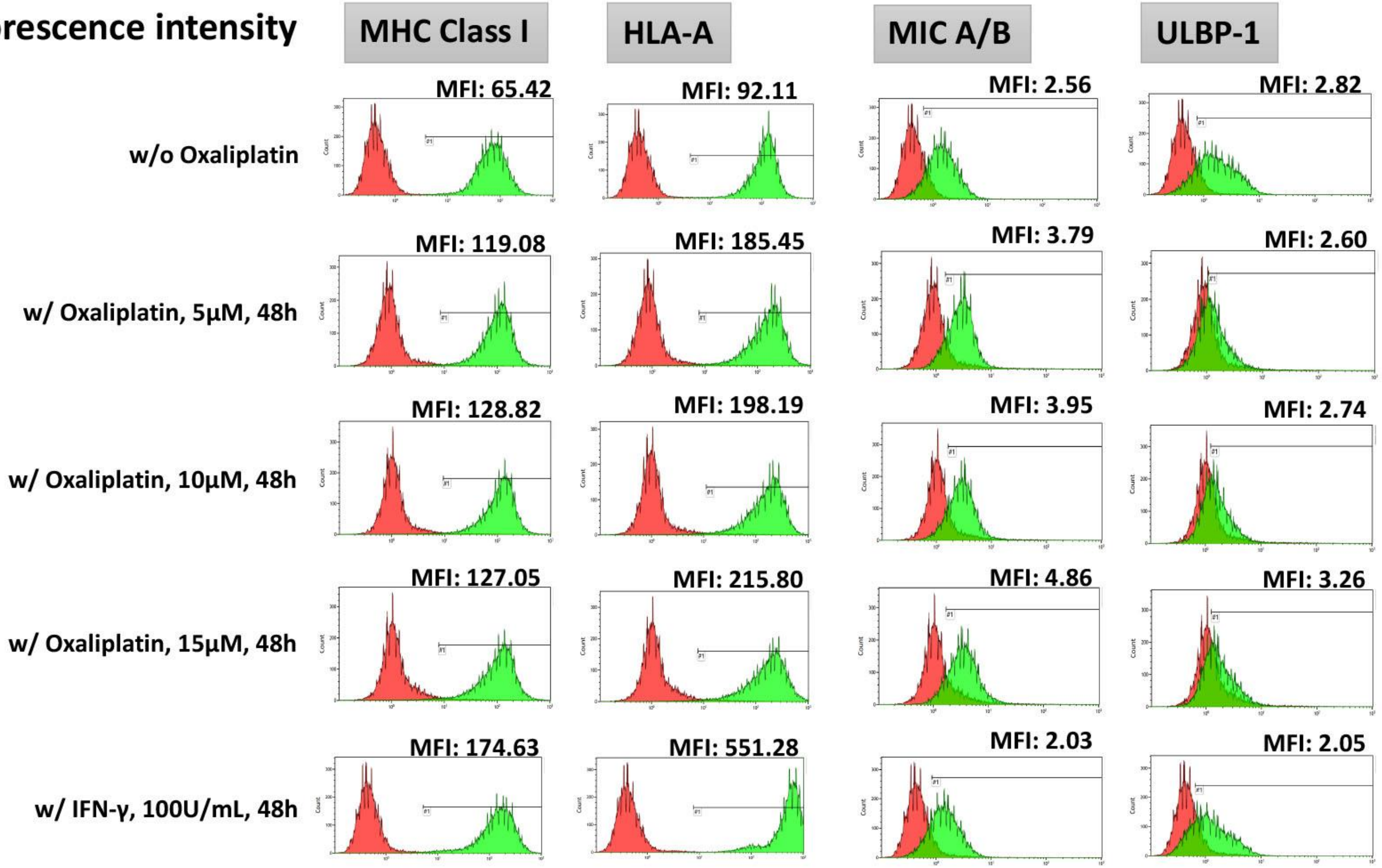

Figure 2G, 2H

SW480

5-FU, 48h

MFI : mean fluorescence intensity

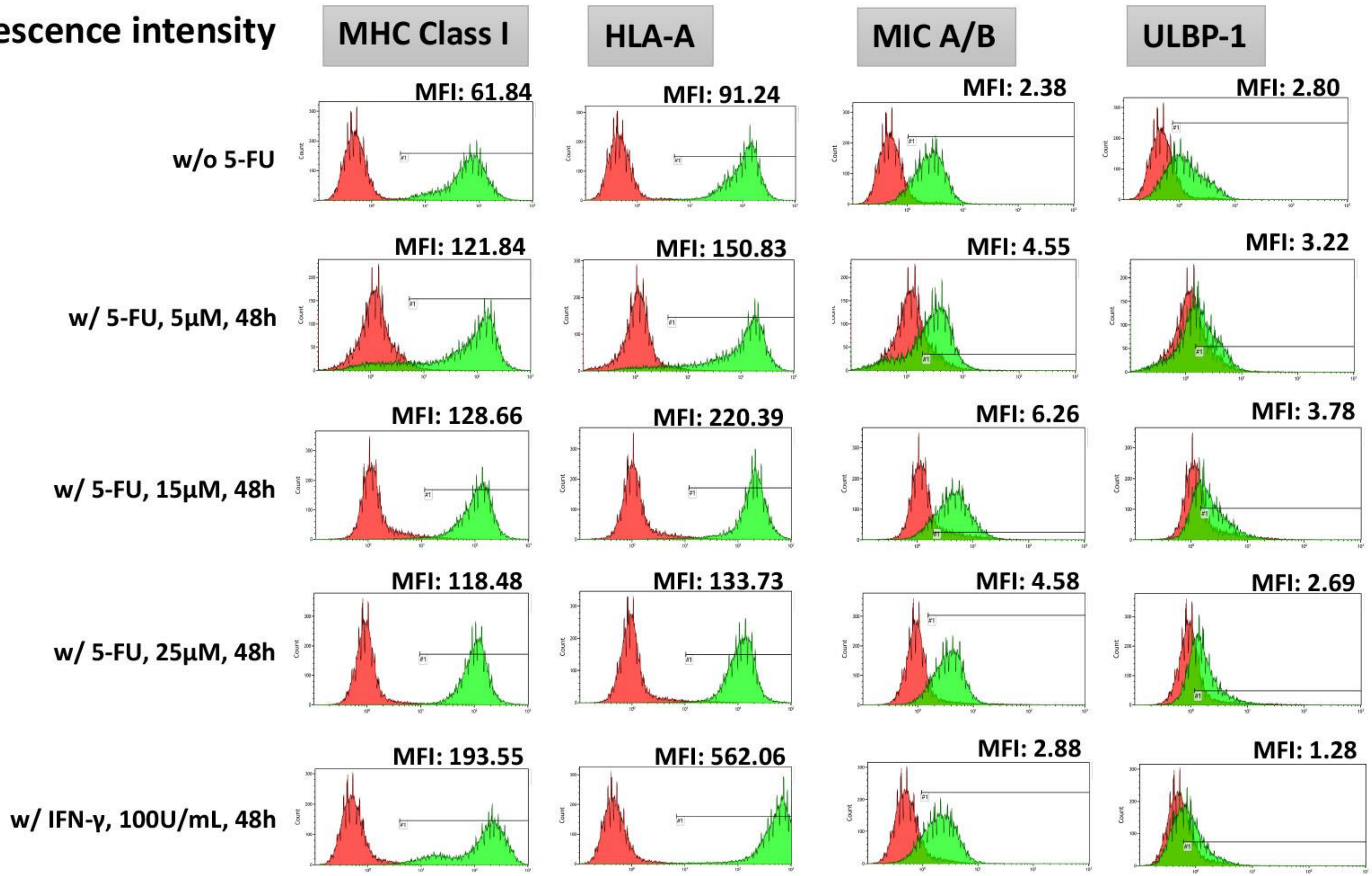

Figure 2G, 2H

SW480

5-FU, 48h

MFI : mean fluorescence intensity

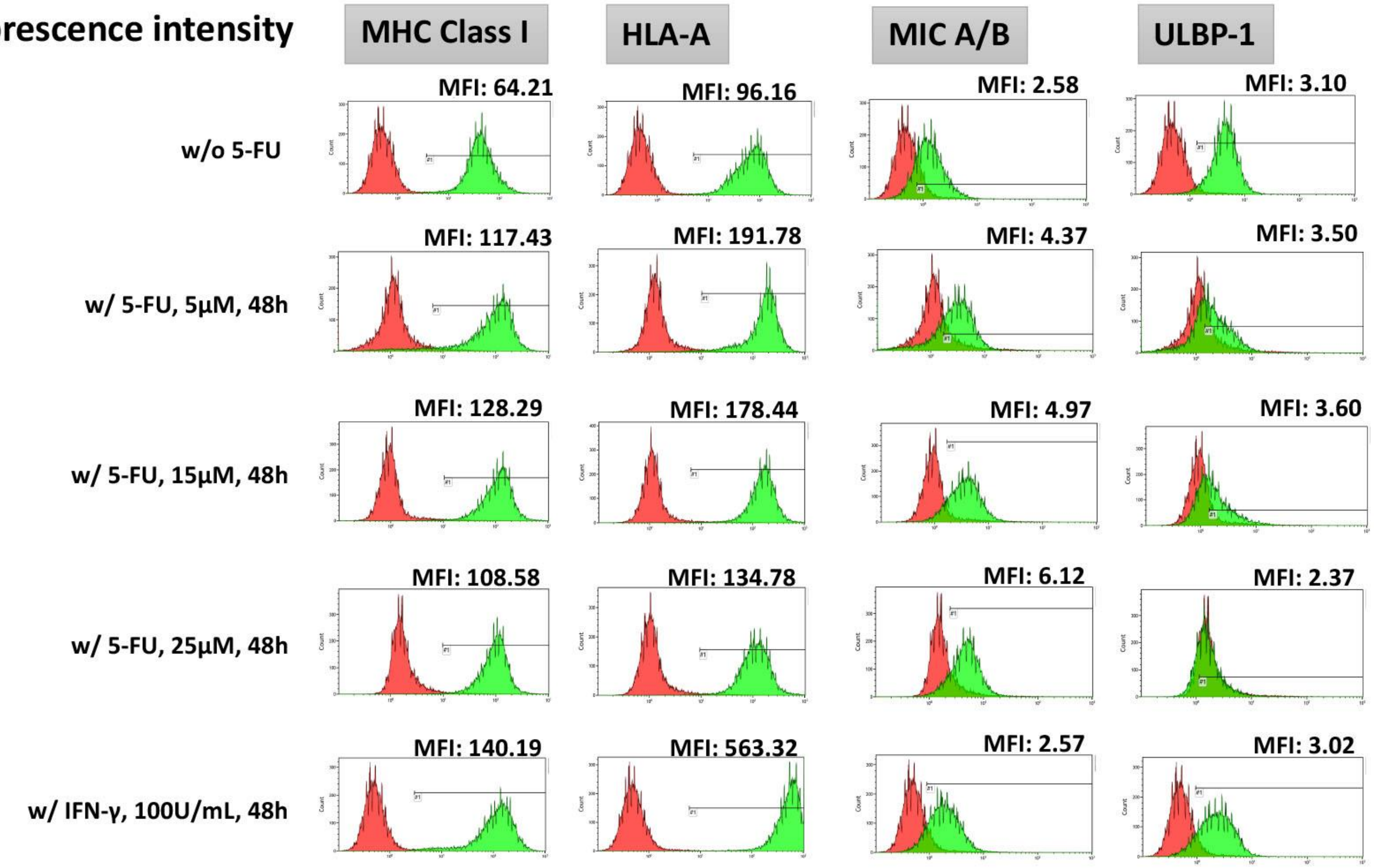

Figure 2G, 2H

SW480

5-FU, 48h

MFI : mean fluorescence intensity

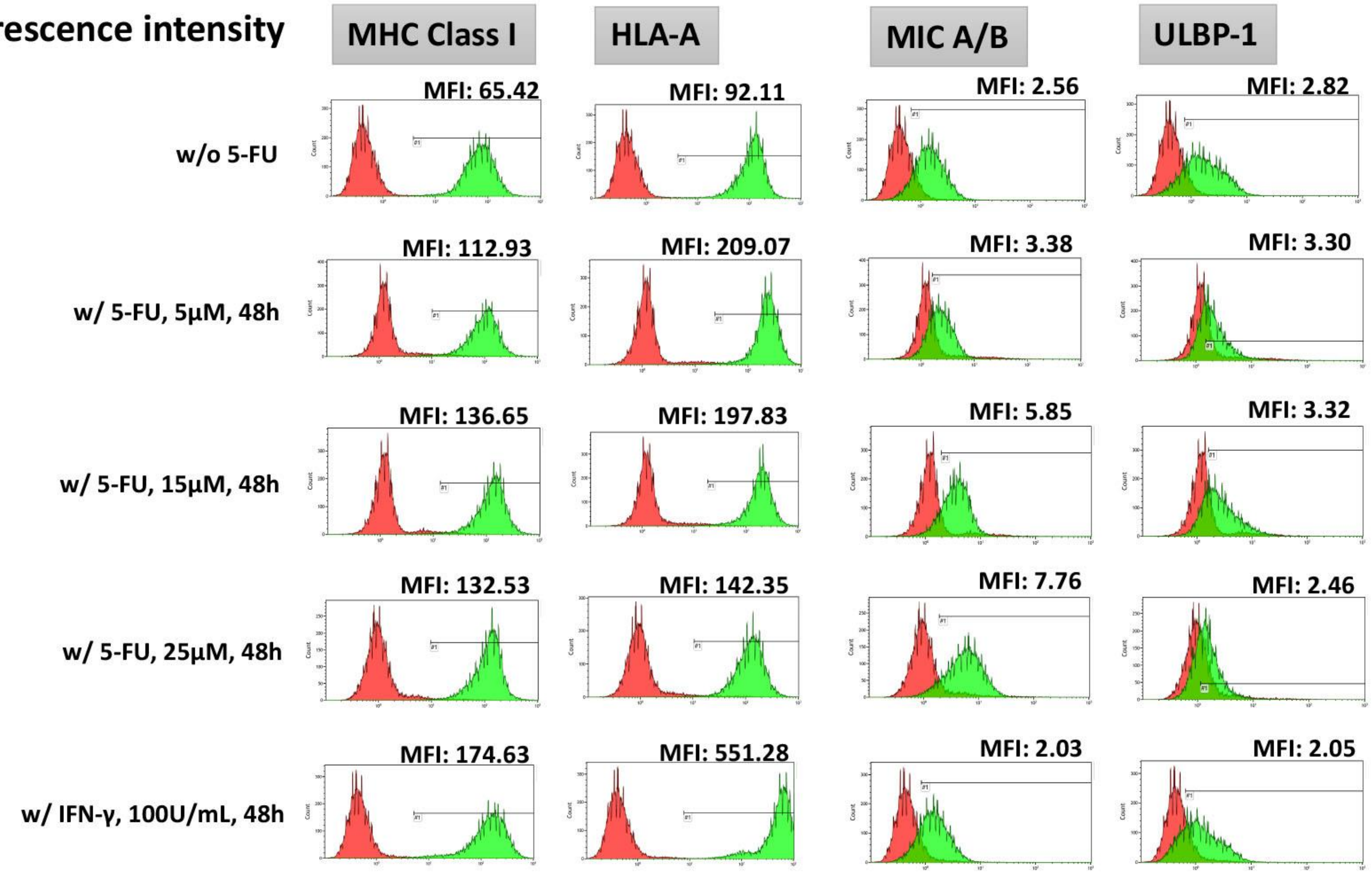

Figure 3A

SW480

IFN-γ : 100U/mL
Oxa : 10μM
SN-38 : 0.1μM
5-FU : 15μM

(48h)

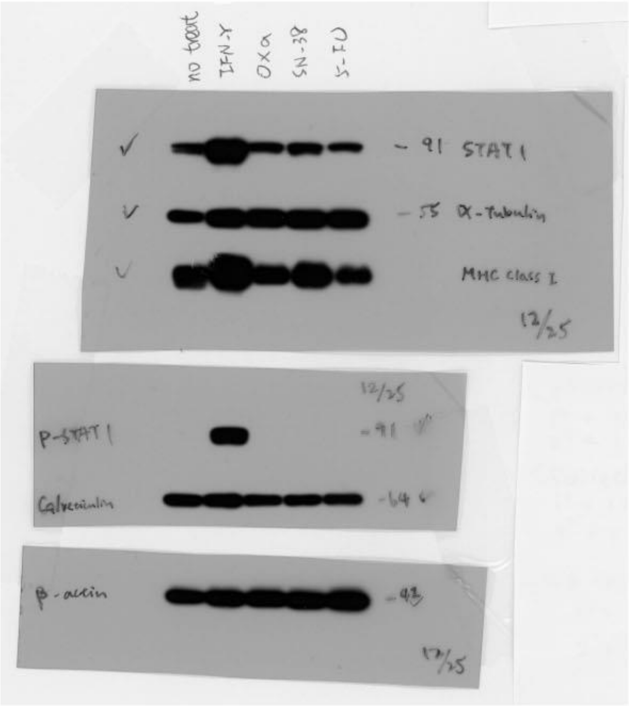

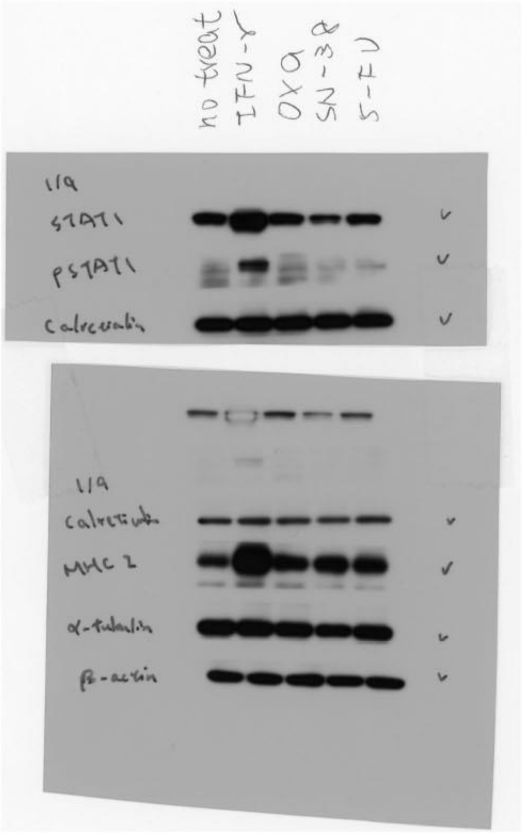

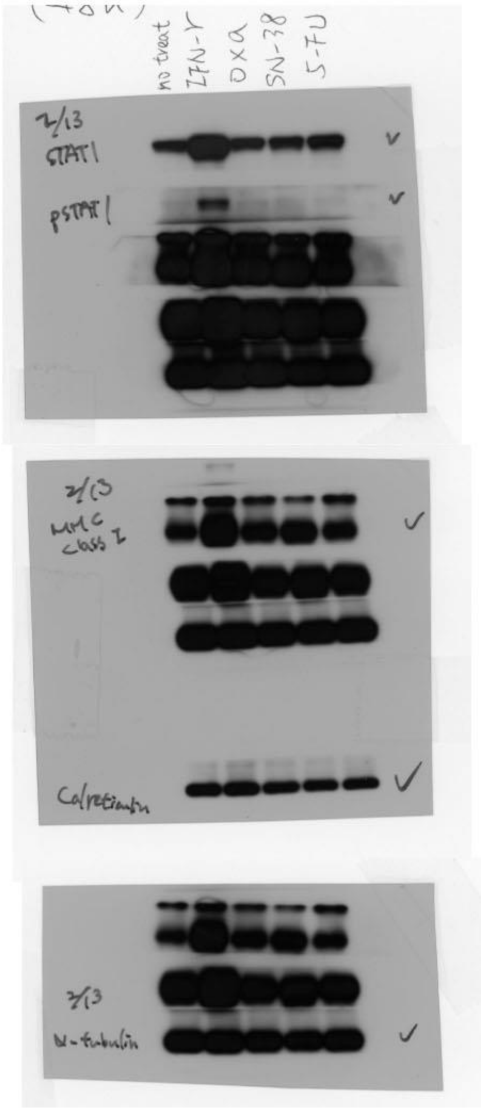

Image acquisition tool : EPSON Perfection V600 Photo
Image processing software : Photoshop (only adjust brightness and contrast)

SW480

Figure 3B

IFN-γ : 100U/mL
Oxa : 10μM
SN-38 : 0.1μM
5-FU : 15μM

(24h)

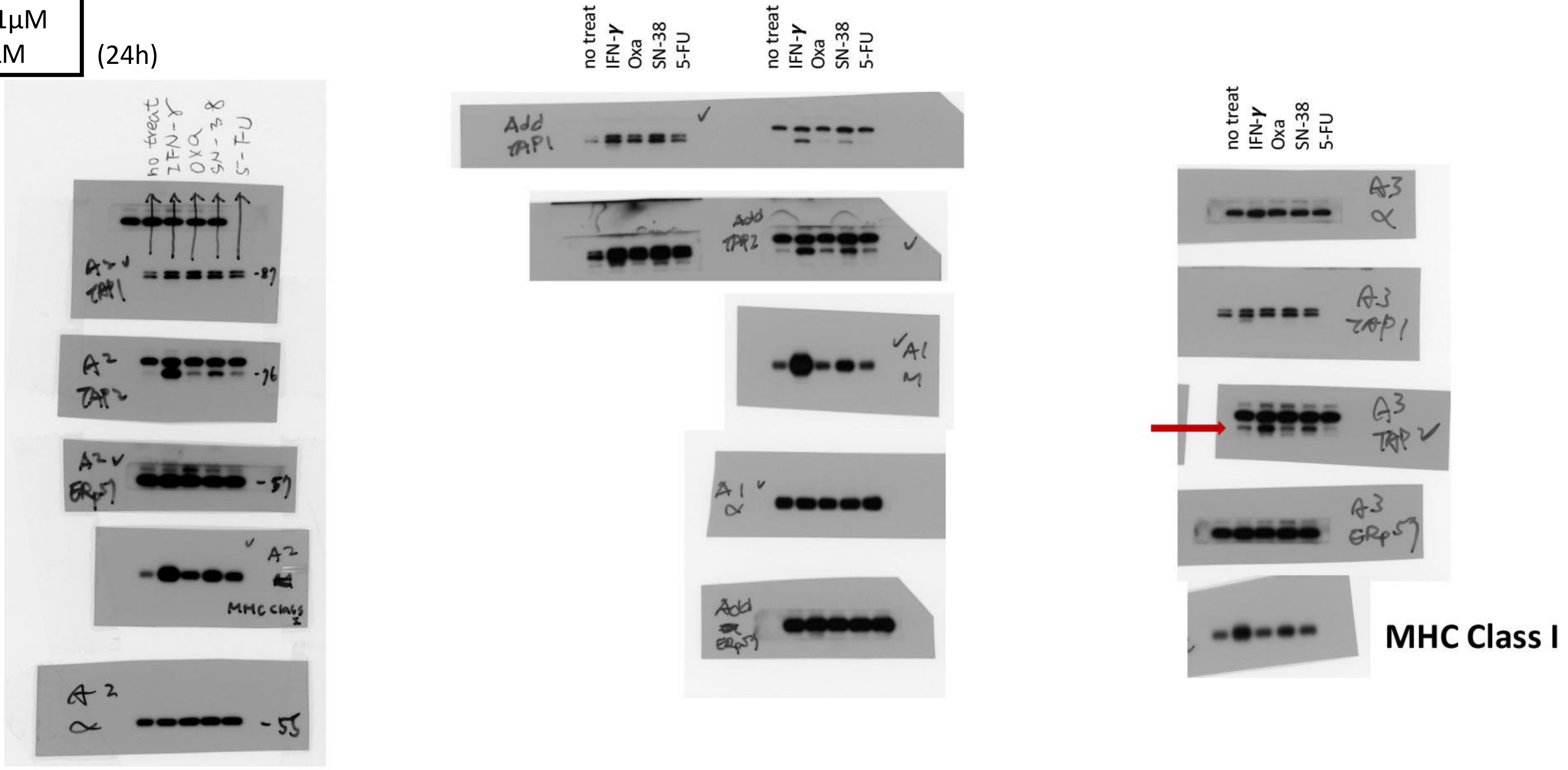

Image acquisition tool : EPSON Perfection V600 Photo
Image processing software : Photoshop (only adjust brightness and contrast)

SW480

Figure 3C

IFN-γ : 100U/mL
Oxa : 10µM
SN-38 : 0.1µM
5-FU : 15µM

(24h)

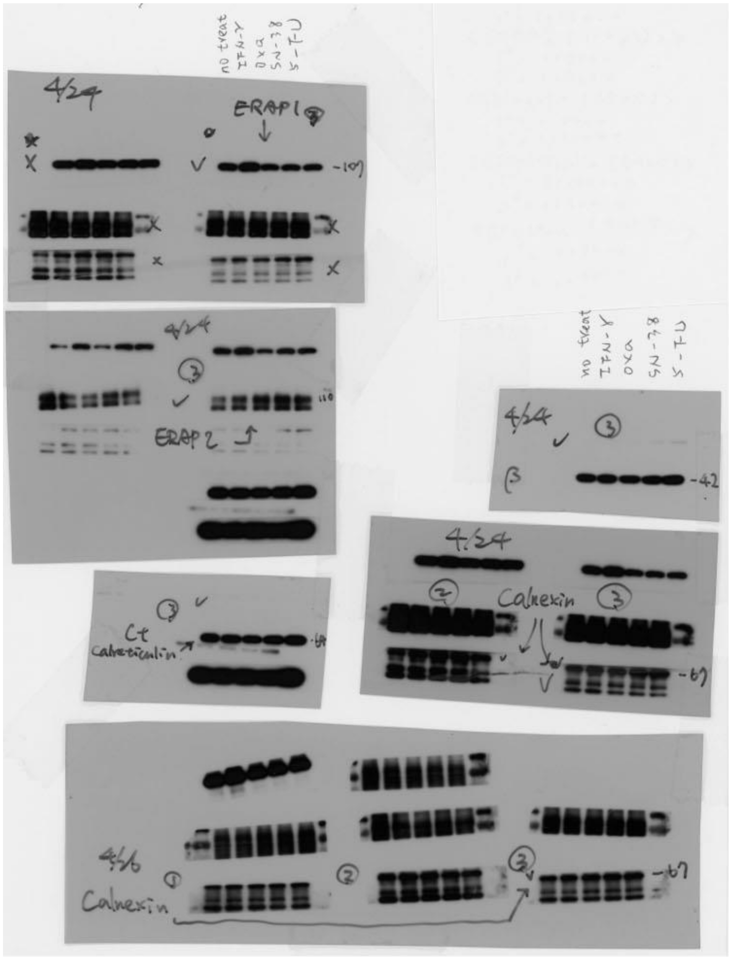

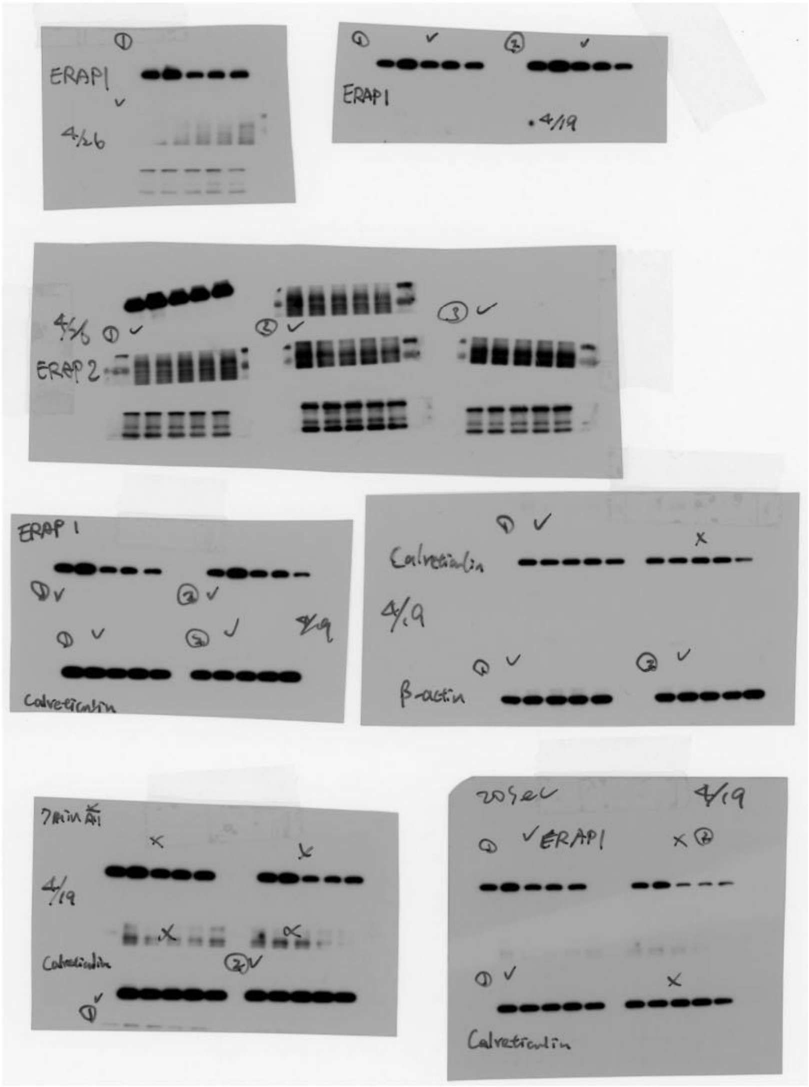

Image acquisition tool : EPSON Perfection V600 Photo
Image processing software : Photoshop (only adjust brightness and contrast)

SW480

Figure 4B

Xfect reagent only
SN-38 : 0.1μM
SN-38 : 0.1μM + Xfect reagent
SN-38 : 0.1μM + Xfect reagent with ICP47 (24h)

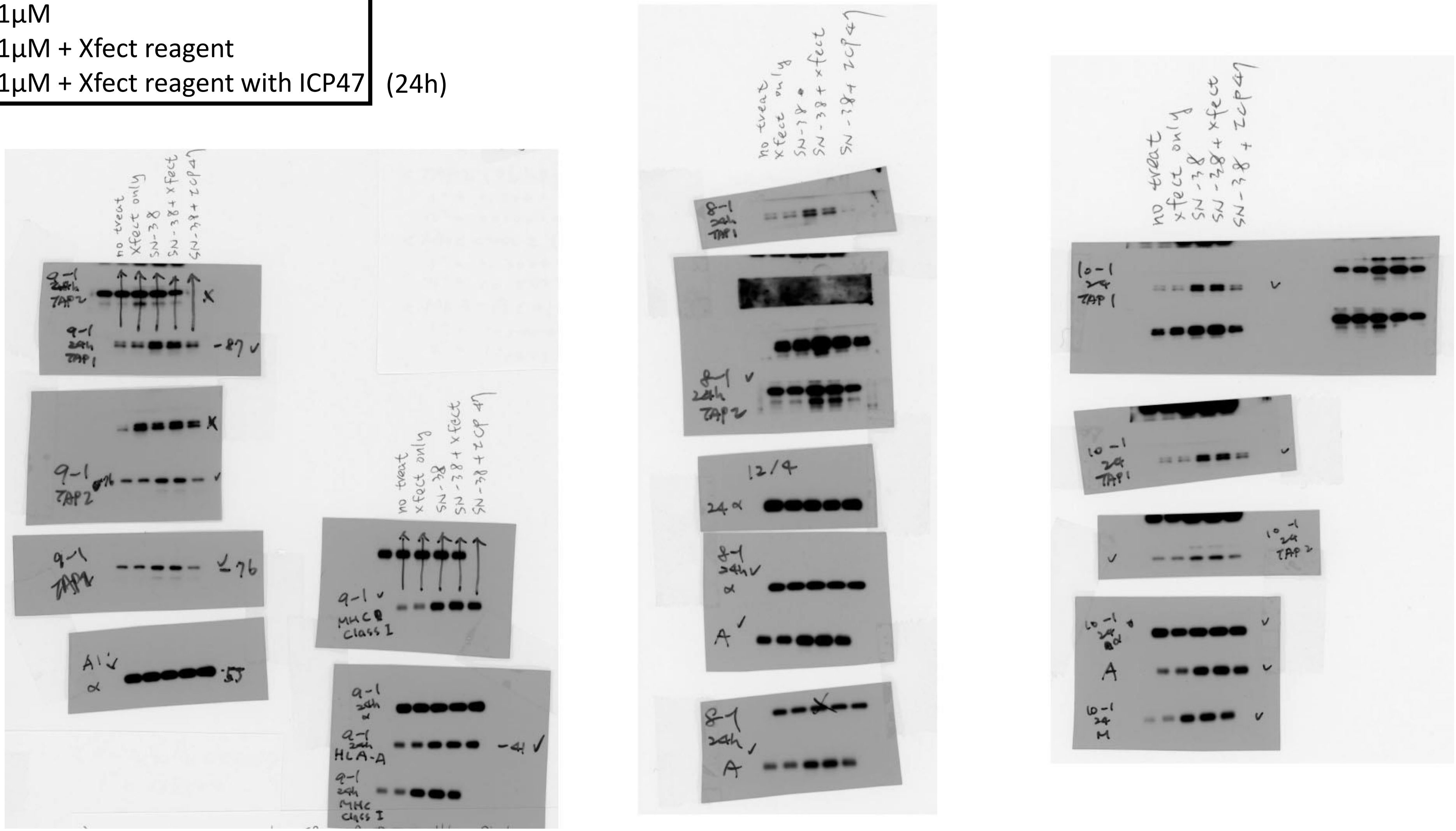

Image acquisition tool : EPSON Perfection V600 Photo
Image processing software : Photoshop (only adjust brightness and contrast)

Figure 5B

Dendritic-cell-based phagocytosis (co-cultured with SW480)

- *human peripheral blood : 50mL*
- *buffy coat : 7.01 x 10^7 cells*
- *$CD14^+$ monocytes : 1.53 x 10^7 cells*

(w/o SN-38)

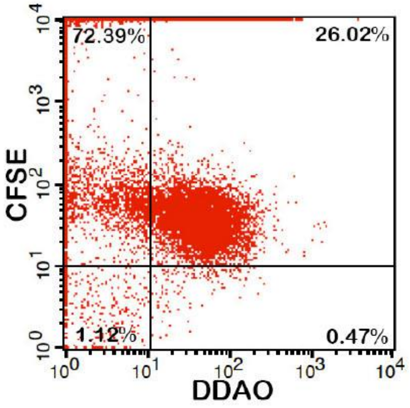

Quadrant Statistics

File: Data.008
Sample ID:
Tube: Untitled
Acquisition Date: 13-Aug-20
Gated Events: 28909
X Parameter: FL3-H (Log)
Quad Location: 11, 10

Log Data Units: Linear Values
Patient ID:
Panel: Untitled Acquisition Tube List
Gate: G1
Total Events: 30000
Y Parameter: FL1-H (Log)

Quad	Events	% Gated	% Total	X Mean	X Geo Mean	Y Mean	Y Geo Mean
UL	20928	72.39	69.76	1.36	1.14	5540.84	2832.68
UR	7523	26.02	25.08	60.24	45.24	612.34	50.12
LL	323	1.12	1.08	3.15	2.32	4.04	3.27
LR	135	0.47	0.45	56.95	40.21	6.66	6.12

(w/ SN-38)

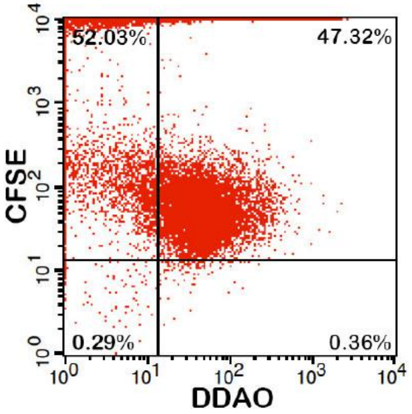

Quadrant Statistics

File: Data.004
Sample ID:
Tube: Untitled
Acquisition Date: 13-Aug-20
Gated Events: 29461
X Parameter: FL3-H (Log)
Quad Location: 13, 13

Log Data Units: Linear Values
Patient ID:
Panel: Untitled Acquisition Tube List
Gate: G1
Total Events: 30000
Y Parameter: FL1-H (Log)

Quad	Events	% Gated	% Total	X Mean	X Geo Mean	Y Mean	Y Geo Mean
UL	15328	52.03	51.09	2.13	1.43	6219.18	3299.51
UR	13940	47.32	46.47	84.89	54.40	2582.47	192.90
LL	86	0.29	0.29	4.45	3.24	6.69	5.77
LR	107	0.36	0.36	49.57	38.42	10.43	9.94
